# Significant fluctuation in the global sulfate reservoir and oceanic redox state during the Late Devonian event

**DOI:** 10.1093/pnasnexus/pgac122

**Published:** 2022-07-30

**Authors:** Chunfang Cai, Chenlu Xu, Mojtaba Fakhraee, Daizhao Chen, Yanyan Peng

**Affiliations:** Key Laboratory of Cenozoic Geology & Environment, Institute of Geology and Geophysics, Chinese Academy of Sciences, Chaoyang District, Beijing 100029, China; College of Earth and Planetary Sciences, Beijing 100049, China; Key Laboratory of Cenozoic Geology & Environment, Institute of Geology and Geophysics, Chinese Academy of Sciences, Chaoyang District, Beijing 100029, China; Guangzhou Marine Geological Survey, Guangzhou 510075, China; Department of Earth and Planetary Sciences, Yale University, 210 Whitney Avenue New Haven, CT 06511, USA; Key Laboratory of Cenozoic Geology & Environment, Institute of Geology and Geophysics, Chinese Academy of Sciences, Chaoyang District, Beijing 100029, China; College of Earth and Planetary Sciences, Beijing 100049, China; Key Laboratory of Cenozoic Geology & Environment, Institute of Geology and Geophysics, Chinese Academy of Sciences, Chaoyang District, Beijing 100029, China

**Keywords:** Frasnian–Famennian (F–F) biotic crisis, Iron speciation, sulfate concentration, S, Fe and P cycling, organic sulfur isotopes

## Abstract

Ocean sulfate concentration might have fluctuated greatly throughout the Earth’s history and may serve as a window into perturbations in the ocean–atmosphere system. Coupling high-resolution experimental results with an inverse modeling approach, we, here, show an unprecedented dynamic in the global sulfate reservoir during the Frasnian–Famennian (F–F) boundary event, as one of the “Big five” Phanerozoic biotic crises. Notably, our results indicate that, in a relatively short-time scale (∼200 thousand years), seawater sulfate concentration would have dropped from several mM before the Upper Kellwasser Horizon (UKH) to an average of 235 ± 172 μM at the end of the UKH (more than 100 times lower than the modern level) as the result of evaporite deposition and euxinia, and returned to around mM range after the event. Our findings indicate that the instability in the global sulfate reservoir and nutrient-poor oceans may have played a major role in driving the Phanerozoic biological crises.

Significance Statement The Late Devonian Frasnian–Famennian (F–F) biotic crisis—one of the “Big five” Phanerozoic mass extinction events—wiped out almost 80% of extant marine invertebrate species, especially low latitude shallow-water faunas and metazoan reefs. The cause of the extinction remains uncertain. Here, results from high-resolution analyses of sulfur isotopes in different sulfur species along with iron speciation and isotope-driven modeling provide evidence for remarkable fluctuation in the oceanic redox state, where the global fixation of pyrite in euxinic environments led to dramatic drop in seawater sulfate level in the beginning of the Upper Kellwasser Horizon, and the ocean was then nutrient-poor and iron-rich during the event, likely hampering any biological recovery.

## Introduction

Sulfate (SO_4_^2−^) as the second most abundant anion after chloride in the modern ocean plays a crucial role in biogeochemical cycles of major elements (e.g. carbon, oxygen, phosphorus, and iron). Despite its overwhelmingly high-modern concentration, oceanic sulfate level is widely viewed to have substantially varied throughout the Earth’s history (1–2), with sub mM range for most of the first 3.5 billion years and several to tens of mM for most of the Phanerozoic Eon (<0.5 billion years ago). Such change in seawater sulfate is conveniently linked to change in the redox state of the ocean–atmosphere system and/or evaporate deposition. Specifically, by promoting microbial sulfate reduction, predominantly anoxic iron–rich conditions throughout most of the Precambrian (>0.5 billion years ago) would have impeded the accumulation of seawater sulfate to high levels, whereas oxygenated Phanerozoic oceans would have allowed the first significant ingrowth in seawater sulfate pool (1). Notable exceptions for oxygenated oceans during the Phanerozoic, however, come during the oceanic anoxic events (OAEs), where pervasive anoxia in the oceans would have led to a rapid decrease in the biodiversity of Earth, broadly referred to as mass extinction events (3,4). With the expansion of euxinic environments in mid- and bottom-water settings, precipitation of micron-scale framboids would have been largely fostered, similar to modern euxinic settings (e.g. Black Sea). Under anoxic to sub-oxic conditions, early diagenetic pyrite would have precipitated below the sediment-water interface. The increased rate of pyrite precipitation would result in isotopically lighter isotopes (^32^S) with seawater sulfate being enriched in heavier isotopes (^34^S). The enhanced rate of microbial sulfate reduction and pyrite formation would, in turn, have resulted in a smaller global marine sulfate inventory with an impact on isotopic compositions of sulfur, the extent of which would depend on other local factors (e.g. sedimentation rate), potentially leading to different isotopic compositions in different parts of the ocean. In concurrence with low oxygen availability, the concentration of seawater sulfate during the mass extinction events is considered to have been low as well (3, 4). The precise quantification of such dynamics in oceanic sulfate level remains, however, uncertain, resulting in an incomplete understanding of the link between large-scale perturbation in the Earth system and chemistry of the ocean during the mass extinction events.

The Late Devonian Frasnian–Famennian (F–F) biotic crisis—one of the “Big five” Phanerozoic mass extinction events, wiped out almost 80% of extant marine invertebrate species, especially low latitude shallow-water faunas, and metazoan reefs (5), or 16% to 20% of genera (6), although such mass extinction events have been considered as a continuous decline of biodiversity throughout the middle to late Devonian at a higher resolution of analysis (60 thousand years) (7). The cause of the extinction is debated, but it has been attributed to changes in climatic dynamics (8–10), oceanic anoxia (4,11), eutrophication, and/or volcanic/hydrothermal activity (12). While there is a relative consensus on the presence of ocean anoxia during the event, the nature of anoxia is not well constrained, in part, due to the lack of high-resolution geochemical data. Generally, the anoxic condition can be categorized into ferruginous (iron-rich) or euxinic (sulfide-rich) conditions, with important implications on nutrient cycling and global cycling of bio-essential elements. For instance, while euxinic conditions hamper the recycling of trace metal micronutrients, it promotes the recycling of phosphorus—as a major limiting factor on oceanic primary productivity. Oppositely, trace metal may not be as efficiently removed under ferruginous conditions, but recycling of phosphorus may be less efficient, owing to the strong association of P with Fe minerals (13). An accurate reconstruction of the redox state of oceans during the Frasnian–Famennian boundary (FFB) is thus a crucial step toward understanding the cause(s) of the F–F mass extinction.

Herein, we couple high-resolution experimental data with an inverse modeling approach to offer a more detailed picture of the change in oceanic chemistry during the event, and use that knowledge to quantify the dynamic in seawater sulfate concentration during the FFB event. We show an unprecedented fluctuation in the ocean chemistry as well as the global sulfate reservoir within several hundred thousand years with substantial impacts on global biogeochemical cycles of elements and marine biology.

### Geological settings and approaches

**Fig. 1.**
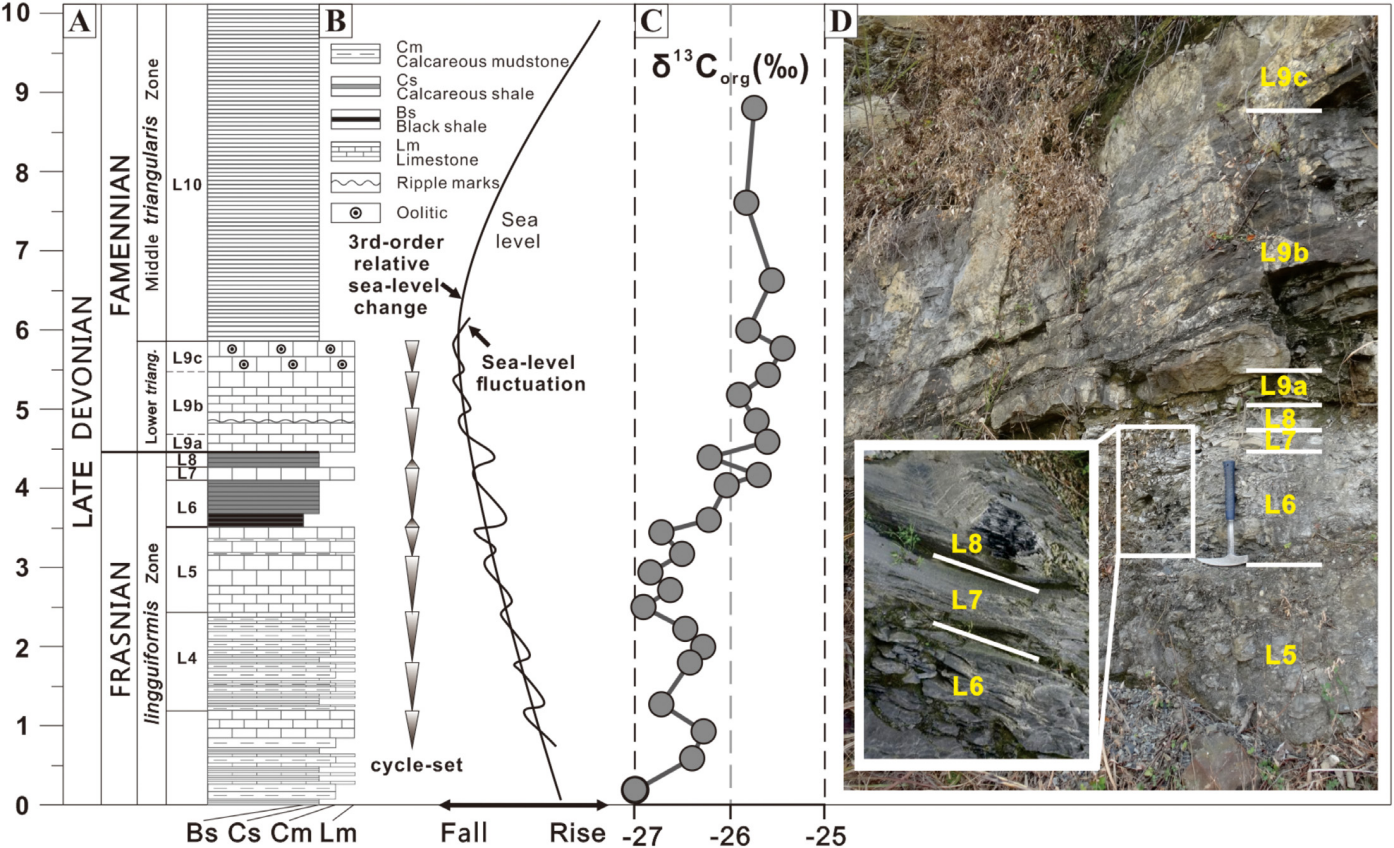
Diagrams showing, (A) lithological column; (B) depositional cycles and relative sea-level changes (third order), and sea-level fluctuations, modified from Ma and Bai (2002; 14) and Zong et al. (2016; 16); (C) organic matter δ^13^C_org_ variation, and (D) field photographs from L5 to L9c sequence.

To reconstruct the oceanic redox state during the FFB extinction, we recruit a combined experimental and modeling approach. A well-exposed Xikuangshan section, Hunan Province, South China (Fig. S1), was chosen for this study and has been systematically studied for biostratigraphy, sedimentology, and chemostratigraphy (14–16) with a positive excursion in δ^13^C_org_ of ∼2‰ across the FFB (Fig. 1A and C). During the Late Devonian, this area was deposited in outer littoral to shallow subtidal zones on the carbonate platform facies. The FFB sequence in the studied area consists of shallow water subtidal mixed carbonate-argillaceous limestone facies, calcareous mudstone, and shales. It contains the Upper *linguiformis* through Middle *Pentagramma triangularis* conodont zones, and shows large scale regression from the Upper *Palmatolepis rhenana* zone to the uppermost portion of the *linguiformis* zone in the latest Frasnian. This regressive sequence is followed by a transgression that starts at the base of the Middle *triangularis* zone. The transgressive sequence is characterized by well-laminated mudstones and shales (upward from the bottom of L10) (14, 15; Fig. 1A and B). Importantly, though the F–F black shale horizon (L6) represents a sea-level rise event in terms of lithologic change, this interval was still deposited in a shallow-water environment (14).

Globally, most studies have revealed that the F–F (Kellwasser) biotic crisis was composed of a series of extinction pulses in conjunction with the deposition of the Lower Kellwasser Horizon (LKH) and Upper Kellwasser Horizon (UKH) (5, 15, 17), i.e. Lower and Upper Kellwasser Crisis. The organic matter rich UKH, found just below the FFB in the uppermost *linguiformis* zone, is the main extinction pulse of the prolonged, stepwise collapse of the Devonian ecosystem (4, 7, 8). The most signifcant biomass and diversity loss occurred at the UKH in the base of the black shale (L6) (15,16), which is similar to some localities in Europe (18, 19). The second step of the UKH mass extinction occurred in the FFB with dramatic reduction of the ostracod and further elimination of brachiopods.

The FFB at the top of bed L8, or the transition from *linguiformis* to *triangularis*, is well defined on the basis of conodonts and the UKH black-shale (16; Fig. 1A). The FFB age was recently estimated at 371.93 to 371.78 Ma through the U-Pb ziron dating supplemented by depositional rate estimation (20), and suggested to be at 372 ± 1.6 Ma by current Geological Time Scale (21). The entire Upper Kellwasser crisis was estimated to last about 0.2 Ma based on high-resolution conodont biostratigraphy and cyclostratigraphy. Thus, *linguiformis* zone was estimated to last about 0.9 to 1.0 Ma (22, 23). The deposition of the Xikuanshan section started at about 372.4 Ma. Thus, each sample can be estimated for its depositional age (Table S1), by assuming that the sediments was deposited at same sedimentary rate before and after UKH, and at the same rate during the UKH.

We first conducted a high-resolution analysis of iron speciation on a suite of sedimentary rocks from the section, wherein the ratios of different iron pools (e.g. pyrite, highly reactive, and total iron) were quantified in order to distinguish between anoxic iron–rich and anoxic sulfide–rich conditions. We further performed sulfur speciation analysis to quantify each pool of sulfur with different oxidation states as well as measured the isotopic composition of each sulfur pool.

The experimental data were, then, used to quantify the seawater sulfate concentration during the Late Devonian FFB event. Taking an inverse modeling approach, our model is based on a well-established mass balance framework for sulfate isotope where the concentration of seawater sulfate through time is controlled by the riverine flux of sulfate (*J*_in_) and its composition value (*δ*_in_), the flux of reduced sulfur as pyrite (*J*_py_), the isotopic difference between seawater sulfate and pyrite (*Δ*), and flux of sulfate evaporites (*J*_ev_) and its isotopic composition (*δ*):

$$\begin{equation*}
\frac{{d\left[ {S{O_4}} \right]{\delta}}}{dt} = \,\,{J_{in}}{\delta _{in}} - \,\,{J_{ev}}{\delta } - \,\,{J_{py}}\left( {\delta - \Delta } \right).
\end{equation*}
$$

The flux of pyrite (*J*_py_) was parameterized so that it varies with seawater sulfate concentration (Fig. S2). The values of *dδ/dt, δ*, and *Δ* and in the model were taken from our measurements. We also used measurements from other parts of the world to provide a more global-scale range for oceanic sulfate concentration (see the “Materials and methods” section). Finally, to account for the uncertainty involved in choosing model parameters, we employed a stochastic approach, in which, instead of using fixed values, the modeling input parameters were randomly sampled within their most reasonable ranges, and assumed a uniform distribution, the most probable range of seawater sulfate concentration during the UKH was obtained (Fig. S2).

## Results

Micron-scale pyrite framboids were observed to occur randomly and individually as closely packed, spherical aggregates with uniform sizes in the UKH mudstone samples X-19 at L6, X-20, and X-21. The statistics of framboid size distribution show that sample X-19 has a mean framboid diameter of 4.9 μm, a maximum framboid diameter (MFD) of 12.5 μm, and a *P*_T_ (the percentage of ≥10 μm framboids in total) of 3.4% (Fig. S3), whereas samples X-20 has larger mean framboid diameters of 8.0 and 7.8 μm, MFDs of 18.2 and 18.6 μm, and *P*_T_ values of 22.4% and 17.9%, respectively.

**Fig. 2.**
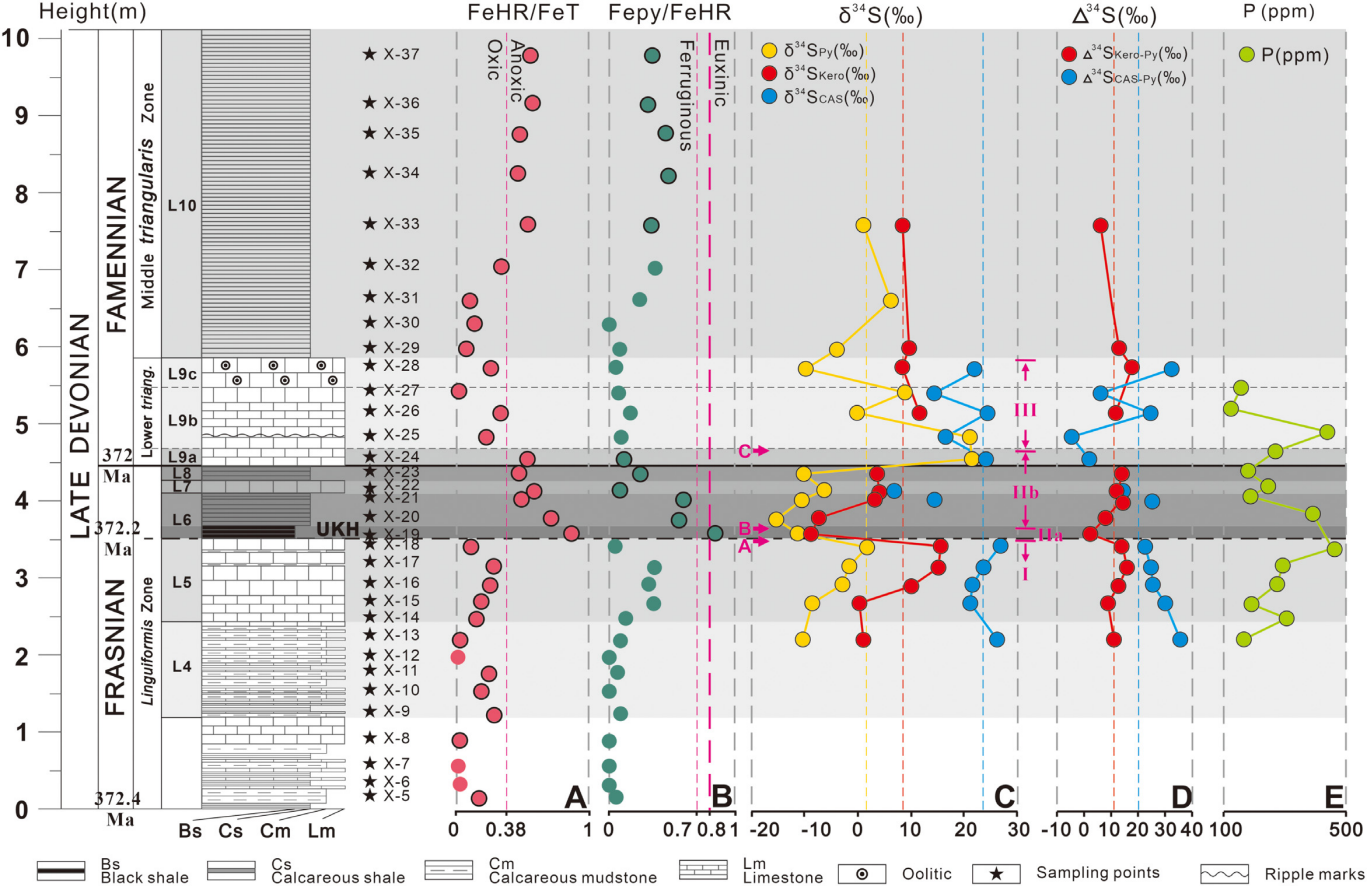
Xikuangshan (South China) FFB section with stratigraphic column and sample number showing, (A) Fe_HR_/Fe_T_; (B) Fe_Py_/Fe_HR_; (C) variations in δ^34^S_Kero_, δ^34^S_CAS_, and δ^34^S_Py;_ (D) variations in Δ^34^S_CAS-Py_ and Δ^34^S_Kero-Py_, and (E) P contents in this study.

Almost all the samples in this study have total Fe (Fe_T_) > 0.5 wt% (Table S2), thus iron speciation can be used to reflect ocean redox conditions by considering the fractional abundance of Fe in highly reactive Fe (Fe_HR_) and pyrite Fe (Fe_Py_) (24). In most modern and ancient sediments deposited beneath anoxic bottom waters, Fe_HR_/Fe_T_ exceeds 0.38, but this threshold value can be reduced to 0.22 for thermally altered ancient sedimentary rocks (25–26). For a euxinic water column, Fe_Py_/Fe_HR_ in the underlying sediments usually exceeds 0.8. For the Xikuangshan section, Fe_HR_/Fe_T_ ratios are 0.11 to 0.29 with the minimum in L5/L6 boundary, and then rise sharply to a maximum of 0.88 in L6, followed by a decrease to 0.54 in L9a (Fig. 2A). This changing trend resembles that of Fe_Py_/Fe_HR_ (Fig. 2B), which increases from 0.03 in L5 to 0.83 in L6, and then decreases to 0.11 in L9a. The Fe_HR_/Fe_T_ ratios of L9b and the lower part of L10 range mainly from 0.04 to 0.25 and are >0.38 at the top of the section. Thus, based on the iron speciation, the sediments across the FFB are further divided into intervals (I, IIa, IIb, and III, Fig. 2A and B). Interval I sediments have Fe_HR_/Fe_T_ < 0.30 and low Fe_Py_/Fe_HR_, interval II has Fe_HR_/Fe_T_ > 0.38 and can be further divided into interval IIa with Fe_Py_/Fe_HR _> 0.7 and interval IIb with Fe_Py_/Fe_HR_ from 0.25 to 0.58, and interval III has Fe_HR_/Fe_T_ ≤ 0.25 and significantly lower Fe_Py_/Fe_HR._ ratios from 0.07 to 0.22.

**Fig. 3.**
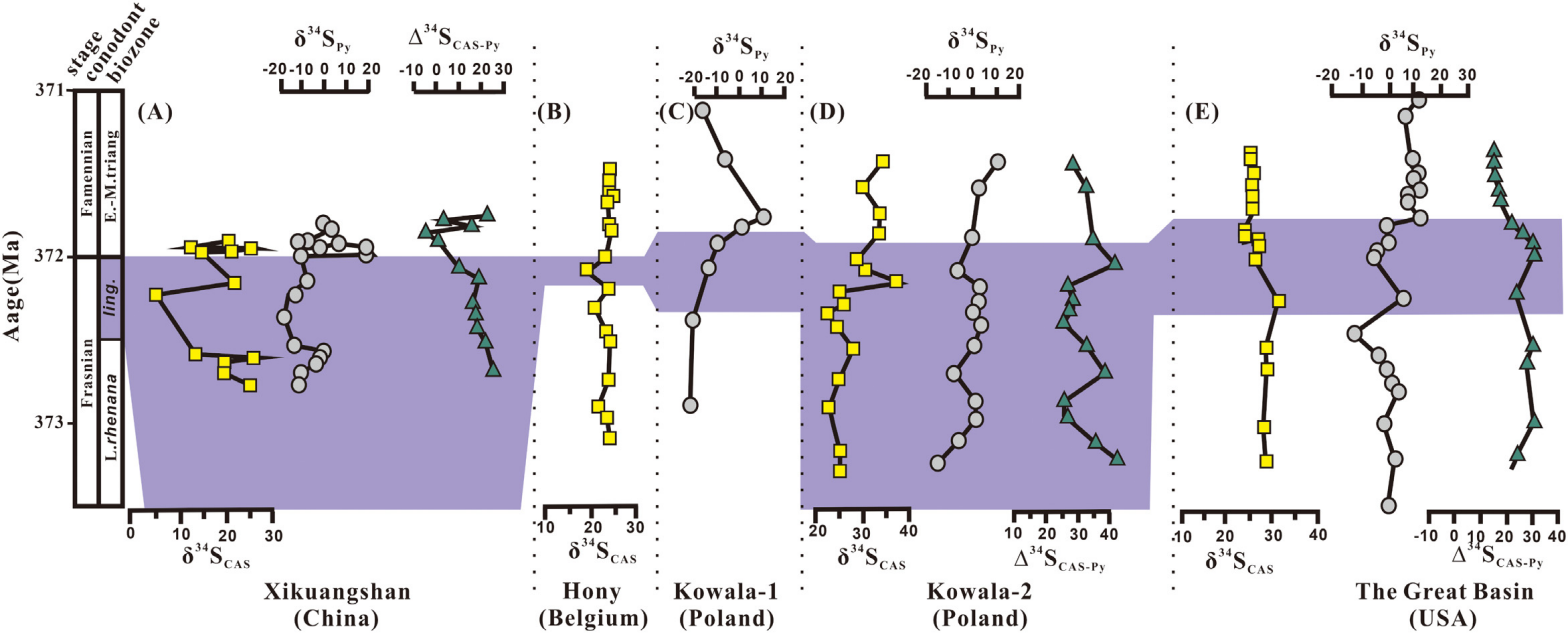
Pyrite and CAS sulfur isotopic compositions across the FFB from, (A) Xikuangshan, China (this study), (B) Hony section from John et al. (28), (C) Kowala-1 from Joachimski et al. (18), (D) Kowala-2 from Chen et al. (30), and (E) the Great Basin sections from Sim et al. (4). The shaded area indicates the linguiformis conodont biozone, and the Upper Kellwasser positive δ^13^C excursion begins in the uppermost part of this biozone for all these sections. Abbreviations: L. rhenana, Late rhenana; ling., linguiformis; E.-M. triang., Early to middle triangularis.

Isotopic compositions of sulfur species show wide ranges from −9.3‰ to +15.4‰ for organic sulfur (δ^34^S_Kero_), +7.1‰ to +26.2‰ for carbonate-associate sulfate (δ^34^S_CAS_), and −15.2‰ to +22.0‰ for pyrite (δ^34^S_Py_). Within the section, there are three dramatic transitions (A, B, and C) (Fig. 2C). Samples from L5 (interval I) show synchronous positive shifts in the δ^34^S value of the three sulfur species and a gradual decrease in Δ^34^S_CAS-Py_ value (Fig. 2D). Both δ^34^S_Kero_ and δ^34^S_Py_ show an abrupt decrease from 15.7‰ to −9.3‰ and from 2.3‰ to −11.4‰, respectively, at transition A across the L5–L6 boundary. Interestingly, the isotopic difference between organic sulfur (kerogen) and pyrite (∆^34^S_Kero-Py_ = δ^34^S_Kero_–δ^34^S_Py_) shows a correspondingly sharp fall from 13.3‰ to 2.0‰ (Fig. 2D). At transition B in the interval IIa, δ^34^S_Kero_ and Δ^34^S_Kero-Py_ show coupled positive shifts alongside a decrease in δ^34^S_Py_. Further upwards within interval IIb, δ^34^S_Kero_, δ^34^S_Py_, and Δ^34^S_Kero-Py_ show increasing trends, and δ^34^S_CAS_ shows a decrease towards the FFB. Across the FFB, there is a dramatic positive excursion of δ^34^S_Py_ with a magnitude of 32.3‰ in this study (Fig. 2C). There exists a negative relationship between Fe_Py_/Fe_HR_ ratio and Δ^34^S_Kero-Py_ (Fig. S4). Similar negative shifts in δ^34^S_CAS_ and positive shifts in δ^34^S_Py_ leading up to the FFB followed by dramatic positive shifts after the FFB occur at sections in the Poland (18, 27), Belgium (28), and the Great basin, United States of America (4) (Fig. 3), suggesting that this sulfur isotope trend is a global signal, although the magnitudes are different—partially due to the different sample resolutions. Within interval III, which follows transition C at the FFB, δ^34^S_CAS_ and δ^34^S_Py_ show fluctuations and a decoupled changing trend while δ^34^S_Kero_ keeps relatively stable.

## Discussion

### Preservation of geochemical signals

At Xikuangshan, the pyrite contents in the CAS extracted samples are mostly <0.6 wt.%. Among other four samples with pyrite contents >2.0 wt.%, only a sample with pyrite content of 3.4 wt.% was measured for both δ^34^S_CAS_ and δ^34^S_Py_, but shows high Δ^34^S_CAS-Py_ of 25.0‰ (Table S2). More importantly, neither pyrite content nor δ^34^S_Py_ is correlated significantly with δ^34^S_CAS_ values (Fig. S5A). The two lines of evidence indicate no obvious influence from pyrite oxidation during experiment processing, outcrop weathering, or secondary atmospheric sulfate in our study (4, 29–32). Notably, the δ^34^S_CAS_ values from X-24 and 25 are similar to those of δ^34^S_Py_ (Fig. 2C and D), which seems to be related to some contamination from pyrite. However, the negative Δ^34^S_CAS-Py_ value supports well-preserved primary marine δ^34^S_CAS_ signatures as the result of higher δ^34^S_CAS_ than δ^34^S_Py_ in general. The samples were also not significantly altered by, (1) thermal maturation of organic matter as supported by low TOC contents (mostly <0.9%), all the kerogen H/C molar ratios > 0.2, and no relationships of both H/C molar ratios and TOC values to the δ^13^C_org_ values (Table S2, Fig. S5B) (33–35); and (2) the weathering effects as supported by no inverse relationship between Fe_Ox_ and Fe_Py_ (Fig. S5C), because the post-depositional weathering reactions would lead to the loss of pyrite and the increase in Fe_Ox_ simultaneously, thus the inverse relationship between Fe_Ox_ and Fe_Py_ can be expected (36, 37).

### A substantial variation in the oceanic redox state

Our experimental results point toward a substantial variation in the oceanic chemistry in the Late Devonian mass extinction, UKH. More precisely, the results from iron speciation (Fe_HR_/Fe_T_ from 0.29 to 0.11) indicate a switch from a less anoxic to pervasively oxic condition before the event, to extensive anoxia during the event (Fe_HR_/Fe_T_ > 0.38) (Fig. 2A). The change in redox conditions before the event may have resulted in the migration-down of oxic/anoxic boundary and the formation of pyrite and organic sulfur compounds in deeper sediments under more closed conditions with lower sulfate concentrations. Consequently, pyrite, CAS, and organic sulfur show increase in their δ^34^S values (Fig. 2C). While pervasive anoxia during the event has been reported by previous studies (18, 27, 28, 30, 38), our analysis shows a substantial change in the nature of anoxia. Explicitly, the beginning of the events is marked with a widespread sulfide-rich condition from enhanced microbial sulfate reduction (MSR), as indicated by high pyrite content of 6.1%, Fe_HR_/Fe_T_ ratio of 0.88 and Fe_Py_/Fe_HR_ of 0.83 in L6 in interval IIa (Fig. 2A and B), consistent with the biological crisis at this time (9,15). The euxinic condition at the beginning of the UKH is in line with the results from pyrite framboid sizes and the isotope composition of sulfur species as well. Euxinic pyrite framboids have been shown to be smaller in average and less variable in size than framboids from sediments underlying dyoxic and oxic water column (39). A euxinic environment for the deposition of sample X-19 in L6 is indicated by the pyrite framboid mean diameter, MFD and *P*_T_ (39; Fig. S3), and the negative shift in δ^34^S value from +2.3‰ in sample X-18 to −11.3‰ in X-19 during the transition from L5 to L6. Although widespread volcanic and hydrothermal activity during the UKH as indicated by Hg enrichment and isotopic composition (40) may provide isotopically light sulfur, such a source of sulfur is well known to have δ^34^S values heavier than −5‰, and are thus not the main source of sulfur in the X19 pyrite. In contrast, sulfur disproportionation in euxinic environments is the most likely reason to lead to lower δ^34^S values in H_2_S and resulting pyrite. Sulfur isotope is found to be rather exchangeable between dissolved sulfide and proto-kerogen, and under conditions of high dissolved sulfide/organic sulfur ratios, proto-kerogen may have δ^34^S values approaching that of dissolved sulfide and thereby pyrite via isotope exchange (41). The ∆^34^S_Kero-Py_ at the beginning of the UKH shows a minimum value of 2.1 among all the measurement may have resulted from high free H_2_S or euxinic conditions. Such euxinic conditions were, however, ephemeral and replaced by large-scale ferruginous conditions, as shown by Fe_HR_/Fe_T_ ratios between 0.38 and 0.7 in interval IIb (Fig. 2A). This is consistent with the high values of ∆^34^S_Kero-Py_ during this interval, and a negative relationship between Fe_Py_/Fe_HR_ ratio and ∆^34^S_Kero-Py_ value (Fig. S4). The relationship well indicates that, with increasing ∆^34^S_kero-Py_ values, depositional environment is evolved from euxinic conditions towards Fe-rich conditions. This anoxic ferruginous bottom water at the end of L9a was changed to the less anoxic or more oxic as indicated by the decrease in Fe_HR_/Fe_T_ ratio from 0.52 to 0.23, signalling an enhancement in the oceanic oxygen availability, and thus paving the road for the biological recovery in the aftermath of the event. As suggested by the results from iron speciation on the top of the section, this less anoxic condition was again replaced by anoxic ferruginous condition, indicating a highly variable oceanic redox state during and after the UKH.

### Large dynamic in the seawater sulfate level

**Fig. 4.**
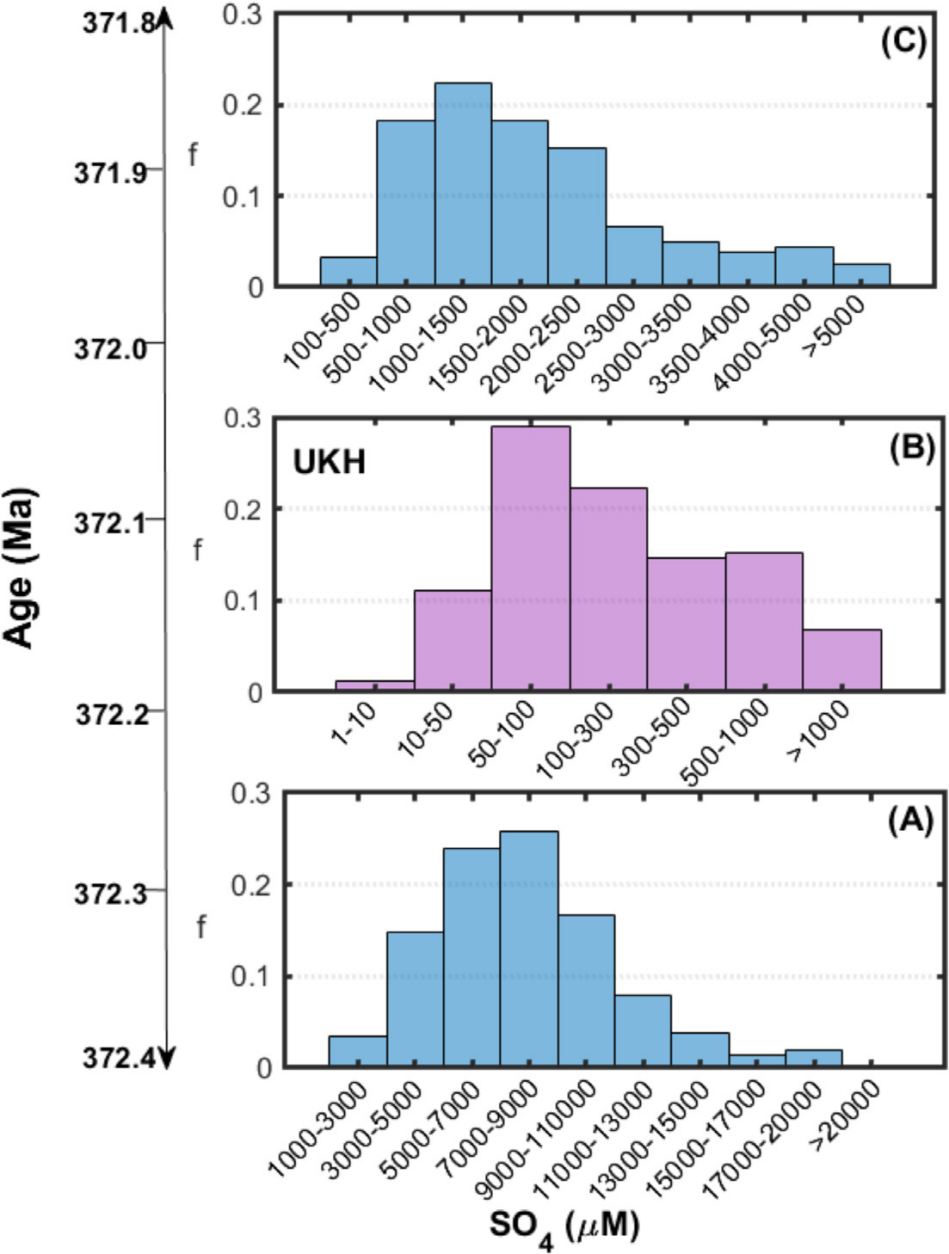
Nonsteady sulfate concentration in the Late Devonian mass extinction event. The histograms are the modeled range of sulfate concentrations for periods of, (A) the interval I before the UKH, (B) the interval II including the sample at the bottom of L9b during the UKH, and (C) the interval III after the UKH. The histograms resulted from the stochastic analysis in which input modeling parameters were randomly sampled within a reasonable range and the most probable range of seawater sulfate concentration was obtained. The radical change in the seawater sulfate concentration during UKH signals a turbulent condition in the ocean–atmosphere system.

Our results from isotope-driven modeling reveal a great fluctuation in the global sulfur reservoir during the UKH. Notably, our modeling results that are informed by our measurements presented herein indicate a significant contraction and expansion in the global sulfate reservoir (Fig. 4). More precisely, based on our results, the concentration of seawater sulfate would have fallen from several mM before the event to 235 ± 172 μM at the end of the event (about two orders of magnitude decrease) in a relatively short geological timescale (∼200 thousand years) and returned to mM range after the event (Fig. 4). The euxinic environment at the beginning of the event would have significantly elevated the sulfide concentration in the ocean, leading to the biological wipe-out, which is also consistent with our Fe speciation results. While the modeled seawater sulfate concentration before the UKH is in line with the suggested Ca-rich seawater with Ca concentrations up to 30 mM and sulfate of ∼9 mM during the Late Devonian based on fluid inclusion analyses (42), our results indicate a much lower sulfate concentration caused by anoxia during the event.

The modeling results are intimately tied with the change in the measured isotopic composition of pyrite (δ^34^S_Py_) and isotopic difference between pyrite and water column sulfate (∆^34^S_CAS-Py_ = δ^34^S_CAS_–δ^34^S_Py_). Mechanistically, MSR occurred in stratified anoxic euxinic water columns, SO_4_ concentrations are expected to decrease if the rate of sulfate reduction is higher than that of its diffusion from the overlying oxic water column, consequently, the δ^34^S values of residual sulfate is expected to increase with the magnitude depending on the proportion of reduced sulfate mass/residual sulfate mass. Sediment IIa shows light δ^34^S_Py_ value, indicating that during the beginning of the UKH, sulfate reduction rate is not much higher than its diffusion and sulfate concentration is still high enough. With the intensive simultaneous evaporative deposition of huge sulfate minerals in Prypiac’ and Dnipro-Donets basins and the Alberta Basin (43–45), and less diffusion of sulfate from the overlying water column, MSR in both the anoxic ferruginous water columns and sediment-water interfaces proceeded to greater degrees and generated more H_2_S precipitating as pyrite as shown by Fe_Py_/Fe_HR_ of about 0.65 despite the absence of free H_2_S in the water. This along with precipitation of massive sulfides such as in southwest Iberia (46) suggests that seawater [SO_4_] was decreased dramatically to the extremely low values, and thus (1) CAS, kerogen, and pyrites become more positive (enriched in ^34^S) in the intervals IIb and across the FFB (Fig. 2C), and (2) precipitant pyrites have δ^34^S values approaching to that of source sulfate, resulting in a small isotopic difference between pyrite and water column sulfate (∆^34^S_CAS-Py_ = δ^34^S_CAS_–δ^34^S_Py_) occurred (2, 47–48).

Building upon this framework, the decrease in the measured ∆^34^S_CAS-Py_ values at the dawn of the event is then suggestive of a contraction in the seawater sulfate pool. The decreasing trend in the measured ∆^34^S_CAS-Py_ at the FFB has also been reported from other sections worldwide (e.g. Fuhe section, Nanhua basin, China; the Great Basin, USA; and Kowala, Poland; Fig. 3) (4, 18, 38), implying a global dynamic in the global sulfate reservoir during this time. The drop in global seawater sulfate is also reflected in anhydrite δ^34^S values. Anhydrites in the *linguiformis* zone have a large range of δ^34^S values from 23.3 to 34.2‰ (*n* = 23), which are similar to worldwide Late Devonian anhydrites from 18.8 to 34.0‰ (*n* = 71) (37). These values are significantly higher than the middle Devonian with an average of about 19‰ (45). It has been demonstrated that sulfate δ^34^S values may have altered due to MSR, sulfide reoxidation and mixing with river-input sulfate, but not by evaporation (2,4, 49). Late Devonian seawater sulfate δ^34^S values show the dramatic increase from the middle Devonian, indicating that a great proportion of seawater sulfate may have been depleted due to MSR in a relatively low sulfate environment after intensive evaporative precipitation of sulfate minerals in several basins. The large change in Late Devonian seawater δ^34^S values must have caused from low sulfate reservoir, and thus any addition of ^32^S-rich sulfate from sulfide reoxidation or river input is expected to lead to significant decrease in total sulfate δ^34^S. In contrast, MSR in low sulfate environments are expected to increase its δ^34^S values. The proposal about low sulfate seawater during the Late Devonian is consistent with our section showing high stratigraphic δ^34^S_Py_ and δ^34^S_CAS_ variability during the FFB event (Fig. 2C).

Our results do not confirm the causal link between the fluctuation in the eustatic sea-level and the pervasive anoxic low-sulfate condition during the UKH. One of the main environmental features in the Late Devonian is the evidence of a large fluctuation in the sea level, with a substantial fall and rise at the FFB (15, 30). By influencing the sedimentation rate and the input flux of nutrient into the ocean, change in sea-level is believed to have modulated the extent of anoxia in the ocean, whereby changing the seawater sulfate level (50, 51). For instance, an increase in the sea-level is considered to elevate the nutrient input into the continental shelves, resulting in higher oxygen consumptions and more anoxia. This, in turn, could lead to an enhancement in the rate of sulfate drawdown through organoclastic sulfate reduction, resulting in an overall lower seawater sulfate level (52). However, our results for an increase in the isotopic composition of pyrite (δ^34^S_Py_) and decrease in the ∆^34^S_CAS-Py_ during the UKH that temporally co-occurs with the low sea-level condition is not consistent with this paradigm, suggesting a minor, if any, role for the sea-level fluctuation on oceanic sulfur cycle. The observed trend in the measured isotopic composition may have, instead, been the result of low-sulfate condition and/or re-oxidation of sulfide (30, 49). Specifically, in the low-sulfate seawaters, the sulfate produced by abiotic oxidation of parent H_2_S is depleted in ^34^S by 4‰ to 5‰, which leads to the ^34^S enrichment in sulfide compare to sulfate, owing to Rayleigh distillation effect (29,30). However, the fraction of H_2_S re-oxidized in reduced environments is considered to be low (0.11 to 0.42) (52), implying an unlikely role of the re-oxidation of sulfide in driving the observed isotopic trend during the UKH. Thus, the low sulfate concentrations during the UKH is the most likely explanation for the decreasing trend in ∆^34^S_CAS-Py_.

### Link of change in seawater sulfate to P contents and biotic crisis

**Fig. 5.**
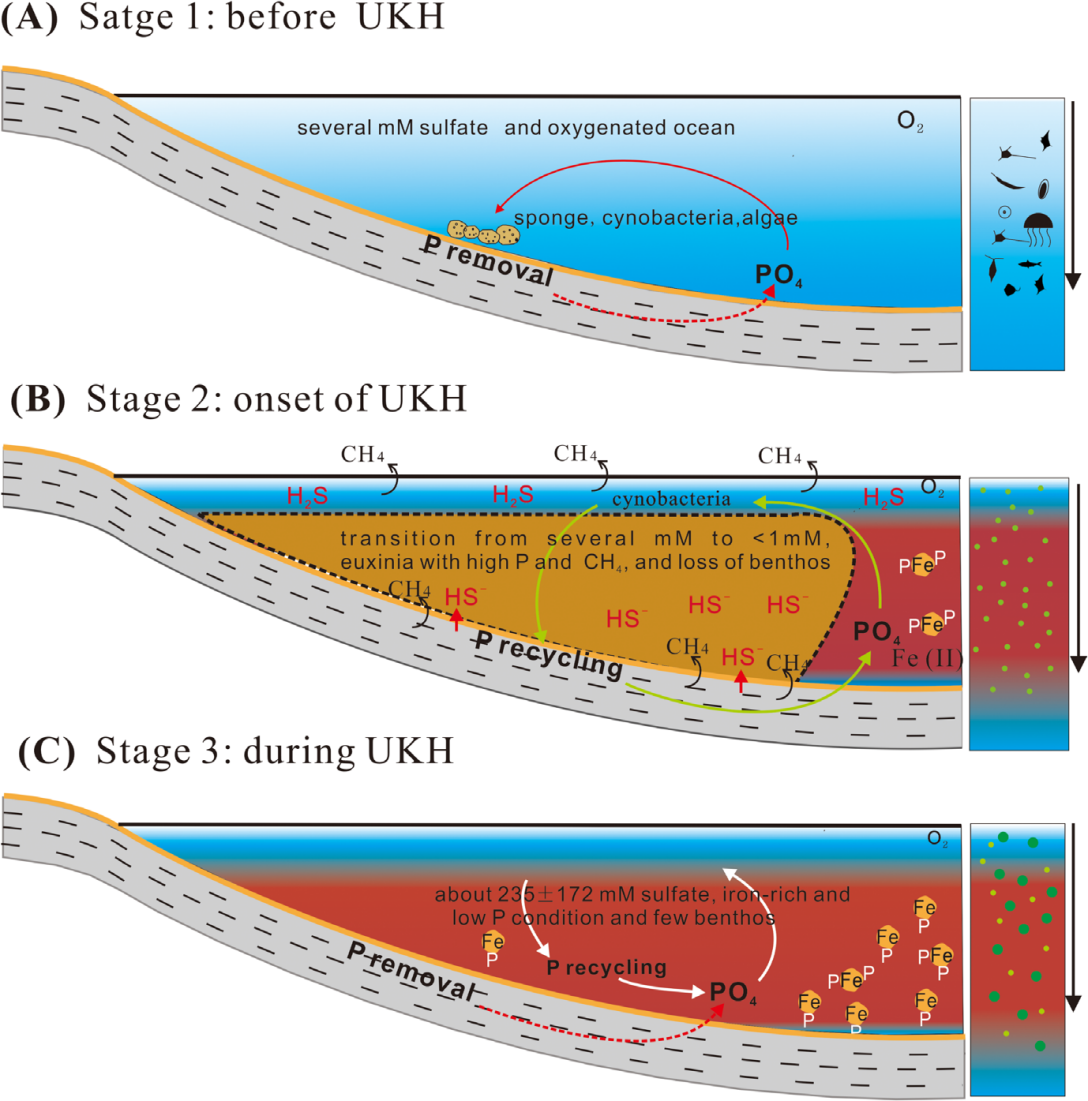
Evolution of seawater sulfate concentrations, redox conditions, and the occurrence of phosphorus and trace elements in the euphotic zone and sediments during the Late Devonian boundary event, UKH.

The great variation in the seawater sulfate level provides insight into the nutrient cycling, and oceanic productivity during the UKH. Specifically, the rate of sulfate drawdown is a function of both organic matter and sulfate availability, and rate of sulfate reduction at mM range is mainly controlled by availability of organic matter (53). Thus, the falling of the seawater sulfate concentration from mM range to less than 500 µM is then indicative of an enhancement in the rate of microbial sulfate reduction, which may have been caused by an elevation in the availability of organic matter. This implies a highly productive ocean with ample macronutrients, most notably, phosphorus. Such conclusion is, indeed, consistent with the measured P data of sediments that show a rise in the P content from 299 to 470 ppm at the beginning of the event (Fig. 2E, Table S1; Fig. S6). The oceanic P concentrations are related to sediment P contents and sedimentation rates, and high P sediments with high sedimentation rates must have deposited from the elevated seawater P concentrations. Coarser sediments prior to the UKH than the UKH sediments suggest, in general, more rapid sedimentation. It can be estimated that UKH sediments were deposited at a sedimentation rate of about one-fourth of sediments before and after the UKH. The higher P sediments show higher sedimentation rates prior to and after the UKH in the FFB sections, suggesting that sediment P contents can well be used to reflect relative changing trends of seawater P concentrations with the highest value at the beginning of the event and the lowest during the UKH (Fig. 2E, Table S1). The rise in P contents at this time is recorded in other sections worldwide such as the Appalachian basin, USA (54) and Steinruch Schmidt, Germany (55), making a case for increased P availability at this time (Fig. S6). Increased P availability can be a result of enhanced volcanic activity during the UKH (12, 56), which would have enhanced the delivery of the nutrient to the ocean (13). A pervasive iron-rich condition during the event, as indicated by Fe speciation results, along with low seawater sulfate concentration point toward a famine ocean with respect to P, which is consistent with low P content at this time (Fig. 5). While such low P and iron-rich conditions may have not been common during the Phanerozoic, it is widely invoked to have prevailed in the Precambrian ocean, where the strong association of P with Fe minerals (13) would have led to a P crisis, resulting in suppressed oceanic productivity and smaller biosphere (57). This environmental condition is suggested to have delayed the emergence of animals as well (58). Taken together as a whole, while the catastrophically chemical transition to sulfidic condition at the beginning of the UKH, might have been the trigger for the widespread biological wipe-out by blood anoxia and olfactory nerve paralysis, the nutrient-poor oceans during the UKH would have likely delayed any biological recovery.

## Conclusions

The catastrophic biological crisis in the Late Devonian would thus indicate a significant and yet unrecognized dynamic in the global biogeochemical cycles of elements. Notably, within several hundred thousand years, the oceanic seawater sulfate concentration would have undergone a large fluctuation with a significant falling during the UKH to about 235 ± 172 μM as a result of oceanic anoxia caused by enhanced oceanic productivity and returning to mM levels after the event. Such dynamic would have extended to nutrient cycling, and oceanic productivity, implying a turbulent condition in the ocean–atmosphere system. A similar dynamic in the redox state and elemental biogeochemical cycles would have likely occurred during the other Phanerozoic anoxic events, yet it suggests that instability in Earth’s surface redox budget may have played an important role in driving the biological crises.

## Materials and methods

### Geochemical analyses

Our outcrop samples were carefully collected from fresh rocks with the weathered surfaces and fracture fillings removed and were powdered and leached using distilled water.

### Analysis of organic matter δ^34^S_Kero_

Organic sulfur δ^34^S_Kero_ is routinely measured on the residual samples after pyrite removal from bulk rocks, which were assumed as pyrite-free (27). Differently, we separated organic matter (or kerogen) from bulk rock and then removed pyrite from kerogen following the method of Cai et al. (59) in this study, and gives an error for the first time (see the supplementary document). Fresh rock samples, after having their weathered surfaces and veins removed, were pulverized as 200 mesh powder (or 74 µm in size) in an agate shatter box. Then, 300 g ground samples were put into PTFE beakers with hot 6 M HCl to dissolve carbonate minerals. The samples were subsequently digested at 60°C for 2 h with excessive amounts of hot 6 M HCl, a 3:1 mixture of 6 M HCl and HF, and then 6 M HCl. After dilution by distilled water and centrifugation, the kerogen was separated from the residue using heavy liquids (KBr + ZnBr_2_) with densities of 2.1 g cm^–3^. Pyrite was removed from the kerogen by adding a mixture of hot 6 M HCl and CrCl_2_ to the ground dry kerogen under a nitrogen cushion with gas flow carrying the H_2_S to a trap containing AgNO_3_. Excess acids and acid soluble salts were removed from the residual kerogen by water washing. About 2 h later, the residual kerogen was collected and reground to expose new pyrite surfaces, and the whole procedure was repeated once more. H_2_S release during this treatment indicates that additional treatment is necessary. After the treatments, a given weight of kerogen (*M*_Kero_, g) was put into a Parr bomb apparatus with deionized water in the bottom, and then combusted at ∼25 atm pure oxygen to oxidize organically bound sulfide and any pyrite remaining pyrite contamination to sulfate and iron oxides. The dissolved sulfate and iron oxides were then diluted to ∼200 mL with deionized water, and acidified to pH < 2, 10 mL of which was used to measure the dissolved iron content (*C*_Fe_, ppm) using atomic absorption spectrometry in order to determine the maximal residual pyrite content in the kerogen after the chromium reduction. The residual dissolved sulfate was precipitated as BaSO_4_ (weighed *M*_BaSO4_, g) by adding 20 mL10% BaCl_2_ after oxidation treatment by bromine water. Only BaSO_4_ from the combustion of kerogen with organic sulfur ≥92% was collected for further δ^34^S_Kero_ analyses.

### Analyses of carbonate-associated sulfate δ^34^S_CAS_ and pyrite δ^34^S_Py_

To release carbonate-associated sulfate (CAS) from calcite for δ^34^S_CAS_ analysis, the samples were dissolved by 0.2 M acetic acid for 2 h at room temperature instead of hydrochloric acid (31, 32) to minimize the contamination from pyrite and dolomite. The CAS from samples X-19, 20, and 23 and the kerogen from samples X-24 and 25 were not acquired successfully for the extremely low content of calcareous components or organic matters. Pyrite sulfur (S_Py_) was extracted by chromium reduction and precipitated as Ag_2_S. BaSO_4_ and Ag_2_S were then analyzed on a Thermo Finnigan Delta S mass spectrometer, calibrated by a series of IAEA standards. δ^34^S results are relative to the Vienna Canyon Diablo Troilite (VCDT) standard with the reproducibility of ±0.3‰.

### Fe-speciation and P analyses

Fe-speciation was extracted from carbonate-rich samples following the procedures of Clarkson et al. (24, 60). All Fe concentrations were measured via atomic absorption spectrometry on a PinAAcle 900T and the replicate extractions gave a RSD between 0.2% and 2% for all steps. Sediment P contents were determined by X-ray fluorescence (XRF) spectroscopy with the error of about ±5%.

### Organic matter δ^13^C_org_ and element compositions

δ^13^C_org_ vales were measured on a Thermal MAT-253 mass spectrometer after removing the inorganic carbon and expressed in standard delta notation relative to the Vienna Pee Dee Belemnite (VPDB) standard with an analytical error of ≤0.06‰. The H and C contents of total rock and kerogen were analyzed by EURO3000 with an analytical precision of ±0.5% and the TOC was calculated from decarbonated samples. Element P and other elements were analyzed by XRF with an error of ±5%.

All the samples were analyzed at the Institute of Geology and Geophysics, Chinese Academy of Sciences. The results are shown in Tables S1 and S2.

## Isotope-driven model

To reconstruct the oceanic redox state during the FFB extinction, we recruit a combined experimental and modeling approach.

### Inverse model for seawater sulfate concentration

To estimate the changes in size of the seawater sulfate reservoir, we recruited an inverse modeling approach wherein measured isotopic composition of pyrite and sulfate was used to reconstruct the change in seawater sulfate through time. The model is based on a well-established mass balance model for sulfate concentration and its isotopic composition:

(1) \begin{equation*} \frac{{d\left[ {S{O_4}} \right]}}{{dt}} = \,\,{J_{in}} - \,\,{J_{ev}} - \,\,{J_{py}}, \end{equation*}

(2) \begin{equation*} \frac{{d\left[ {S{O_4}} \right]{\delta}}}{{dt}} = \,\,{J_{in}}{\delta _{in}} - \,\,{J_{ev}}{\delta} - \,\,{J_{py}}\left( {\delta - \Delta } \right). \end{equation*}

Here, *J*_in_, *J*_ev_, *J*_py_, *δ*_in_, *δ*, and *Δ* denote the riverine flux of sulfate, sulfate evaporite flux, the flux of pyrite, the isotopic composition of riverine sulfate, the isotopic composition of seawater sulfate, and the isotopic difference between seawater sulfate and pyrite (∆^34^S_CAS-Py_ = δ^34^S_CAS_–δ^34^S_Py_). The flux of pyrite (*J*_py_) was parameterized so that it varies with seawater sulfate concentration (Fig. S2). The values of *dδ/dt, δ*, and *Δ* and in the model were taken from our measurements.

Given that *dSδ/dt* = *δ* × *dS/dt* + S × *dδ/dt*, the equation (2) can be rewritten using the equation for sulfate concentration mass balance:

(3) \begin{equation*} \left[ {S{O_4}} \right]\frac{{d\delta}}{{dt}} + {\delta}\left( {{J_{in}} - \,\,{J_{ev}} - \,\,{J_{py}}} \right)\,\, = \,\,{J_{in}}{\delta _{in}} - \,\,{J_{ev}}{\delta} - \,\,{J_{py}}\left( {\delta - \Delta } \right). \end{equation*}

Rearranging the equation to solve for sulfate concentration, [SO_4_], we have

(4) \begin{equation*} \left[ {S{O_4}} \right]\,\, = \,\,\frac{{{J_{in}}({\delta _{in}} - \delta )\,\, + \,\,{J_{py}}\left( \Delta \right)\,\,}}{{\frac{{d\delta}}{{dt}}}}. \end{equation*}

*J*_py_ can be parameterized as follows

(5) \begin{equation*} {J_{py}} = \frac{{TOC}}{{TO{C^*}}}RC\,\,\frac{{\left[ {SO_4^{2 - }} \right]}}{{\left[ {SO_4^{2 - }} \right] + k}}.\,\,{A_{area}}.\,\,f, \end{equation*}

Where *R*_C_, *k, A*_area_, and *f*, correspond to, respectively, the maximum sulfate reduction in the modern ocean, half-saturation constant for sulfate uptake by sulfate-reducing bacteria (SRB), area of ocean that sulfate reduction occurs (which is the total area of ocean times a factor less than 0.2, where maximum value correspond to sulfidic condition, Fe_py_/Fe_HR_ > 0.8, Table 1 and S2), and fraction of sulfide ends up in pyrite pool (which is less than 10% for modern and between 10% and 60% for anoxic condition). To account for variability in the oceanic productivity and its effect on the maximum sulfate reduction, we multiply the R_C_ by an organic matter availability factor, which is the ratio of measured TOC in each sample to the typical modern TOC (TOC*). The range of TOC* in the model is assumed to be between 0.5 and 1.5, consistent with the observation in the modern marine sediments (65,66). The parameter *f*_1_ as the fraction of the ocean that is overlain by euxinic condition was calculated by considering the Fe_Py_/Fe_HR_ of each sample, the threshold for sulfidic vs ferruginous condition (Fe_Py_/Fe_HR_ = 0.7), and on the maximum value of 0.2. For instance, the range of *f*_1_ used in the stochastic analysis for the Fe_Py_/Fe_HR_ of 0.83 at the onset of UKH was considered to be between 0.17 and 0.2, which “0.2” is the maximum for the sulfidic condition and “0.17” is calculated based on the maximum of 0.2 and Fe_Py_/Fe_HR_ value of 0.7, as the threshold for the sulfidic versus ferruginous condition. This means that the areal fraction of euxinia for the time corresponding to that sample is between 17% to the maximum of 20% of the total area of the ocean, consistent with the estimates of anoxia during this time using uranium isotope modeling (62). Similarly, for the ferruginous condition indicated by the iron speciation results, the value of *f*_1_ was adjusted based on the maximum value of 0.2, the value of Fe_Py_/Fe_HR_ for each sample, and the threshold for sulfidic versus ferruginous condition (Fe_Py_/Fe_HR_ = 0.7). For example, for the Fe_Py_/Fe_HR_ of 0.58 during the UKH, that is suggestive of ferruginous condition, the range of *f*_1_ for the stochastic analysis for that sample was between 0.14 [0.58*0.2/max (Fe_Py_/Fe_HR_; 0.83)] and about 0.17 (0.7*0.2/0.83). The range of *f*_1_ for each sample is presented in Table S3. As the pyrite burial flux is controlled by the rate of sulfate reduction, we used the well-established framework for quantifying rate of microbial sulfate reduction. This framework describes the rate of enzymatic reaction to be governed by substrate concentrations, which in this case are sulfate and organic matter. The experimental and modeling results show that above certain limit of sulfate, the rate of sulfate reduction is no longer controlled by the concentration of sulfate, but rather is controlled by the availability of organic matter. This is consistent with the observation from modern environments that show the similarity of the sulfate reduction rates in freshwater environments with low sulfate concentration to the marine sediments with 28 mM of sulfate. Experimental studies have also confirmed Monod-type kinetics for rate of sulfate reduction (53, 67).

**Table 1.** Range of parameters for isotope-driven modeling and sensitivity analysis.

**Parameter**	**Symbol**	**Unit**	**Typical range**
Area of the ocean	*A*_area_	m^2^	3.6 × 10^14^
Modern flux of evaporite	J_eva_*	Tmol/year	0.5–2.0 (61)
Riverine flux for sulfate	*J*_in_	Tmol/year	2.5–5.0 (26)
Areal fraction of euxinia	*f*_1_	-	0.01–0.2(62)
Fraction of sulfide as pyrite	*f*_2_	-	0.03–0.5
Isotopic composition of riverine sulfate	*δ*_in_	‰	6–12 (43)
Half-saturation constant	*k*	µM	5–50 (63)
Maximum sulfate reduction rate	*R*_c_	mol/m^2^/year	1–10 (64)
Range of modern TOC	TOC*	%	0.5–1.5 (65,66)

With *dδ/dt, δ*, and *Δ* constrained by this study and other sections worldwide, we calculated non–steady-state seawater sulfate concentration during the UKH. Given the uncertainty involved with estimating some of the parameters, we used a stochastic approach, wherein input parameters were sampled randomly within their expected range (Table 1), and most probable range of sulfate for a given measured *δ* and *Δ* was obtained (Fig. S2).

### Sensitivity analysis of the isotope-driven model

To investigate the sensitivity of our results to the values of model parameters, we conducted a sensitivity analysis. Model parameters were varied within their reasonable ranges (specified in Table 1), and changes in seawater sulfate concentration (ΔSO_4_^2−^) were recorded (Fig. S2). While the results from sensitivity analysis of input parameters fall within the ∼68% CI, it indicates an important role of the riverine flux of sulfate, the isotopic composition of riverine sulfate, fraction of area of the ocean that is euxinic, fraction of sulfide that ends up in pyrite pool, and the maximum sulfate reduction rate (Fig. S2). Notably, increasing the amount and isotopic composition of the riverine flux of sulfate would result in significant accumulation of sulfate, though this significant change may not be consistent with results from the fluid inclusion data (42). The parameters *f*_1_*, f*_2_, as a fraction of area of the ocean that is overlain by euxinic condition and fraction of sulfide produced by sulfate reduction that precipitates as pyrite, exert strong leverage on the non–steady-state concentration of oceanic sulfate. To better constrain the range of values for these parameters, we used our results from iron-speciation, where for Fe_pyr_/Fe_HR_ > 0.8, the value of *f*_1_ is considered to be between 0.1 and 0.2, meaning between 10% and 20% of the area of the ocean is likely covered by the sulfide-rich condition. Values of *f*_2_ were also constrained from our iron-speciation data, where for Fe_HR_/Fe_T _> 0.38, which correspond to anoxic condition, range of 0.1 to 0.4 was taken for *f*_2_, meaning 10% to 40% of the sulfide produced by sulfate reduction would reaction with iron and precipitate as pyrite. The relatively insignificant role of half-saturation constant in determining the non–steady-state sulfate concentration suggests that change in the physiology of sulfate-reducing bacteria may have, unlikely, played a major role in the change of the size of the global sulfate reservoir during the UKH.

## Supplementary Material

pgac122_Supplemental_File

## Data Availability

All data are included in the manuscript and Supplementary Material, including Tables S1 to S5 and Figures S1 to S6.

## References

[1] Halevy I , Peter SE, Fisher WW. 2012. Sulfate burial constraints on the Phanerozoic sulfur cycle. Science. 337: 331–334. 22822147 10.1126/science.1220224

[2] Fakhraee M et al. , 2019. Proterozoic seawater sulfate scarcity and the evolution of ocean–atmosphere chemistry. Nat Geosci. 12: 375–380.

[3] Kump LR , Pavlov A, Arthur MA. 2005. Massive release of hydrogen sulfide to the surface ocean and atmosphere during intervals of oceanic anoxia. Geology. 33: 397–400.

[4] Sim MS , Ono S, Hurtgen MT. 2015. Sulfur isotope evidence for low and fluctuating sulfate levels in the Late Devonian ocean and the potential link with the mass extinction event. Earth Planet Sci Lett. 419: 52–62.

[5] McGhee GR (ed). 2013. When the invasion of land failed: the legacy of the Devonian extinctions. New York (NY): Columbia University Press.

[6] Stanley SM et al. 2016. Estimates of the magnitudes of major marine mass extinctions in earth history. Proc Natl Acad Sci. 113: 6325–6334. 27698119 10.1073/pnas.1613094113 PMC5081622

[7] Fan JX et al. 2020. A high-resolution summary of Cambrian to early Triassic marine invertebrate biodiversity. Science. 367: 272–277. 31949075 10.1126/science.aax4953

[8] Wang X , Liu SA, Wang ZR, Chen DZ, Zhang LY. 2018. Zinc and strontium isotope evidence for climate cooling and constraints on the Frasnian–Famennian (∼372 Ma) mass extinction. Palaeogeogr Palaeoclimatol Palaeoecol. 498: 68–82.

[9] Huang C , Joachimski MM, Gong YM. 2018. Did climate changes trigger the Late Devonian Kellwasser crisis? Evidence from a high-resolution conodont δ^18^O_PO4_ record from South China. Earth Planet Sci Lett. 495: 174–184.

[10] Song HJ et al. 2017. Uranium and carbon isotopes document global-ocean redox-productivity relationships linked to cooling during the Frasnian–Famennian mass extinction. Geology. 45: 887–890.

[11] Bond D , Wignall PB, Racki G. 2004. Extent and duration of marine anoxia during the Frasnian–Famennian (Late Devonian) mass extinction in Poland, Germany, Austria, and France. Geol Mag. 141: 173–193.

[12] Racki G , Rakociński M, Marynowski L, Wignall PB. 2018. Mercury enrichments and the Frasnian–Famennian biotic crisis: a volcanic trigger proved?. Geology. 46: 543–546.

[13] Poulton SW , Canfield DE. 2006. Co-diagenesis of iron and phosphorus in hydrothermal sediments from the southern East Pacific rise: implications for the evaluation of paleoseawater phosphate concentrations. Geochim Cosmochim Acta. 70: 5883–5898.

[14] Ma XP , Bai SL. 2002. Biological, depositional, microspherule, and geochemical records of the Frasnian/Famennian boundary beds, South China. Palaeogeogr Palaeoclimatol Palaeoecol. 181: 325–346.

[15] Ma XP , Gong Y, Chen D, Racki G. 2016. The Late Devonian Frasnian–Famennian event in South China—patterns and causes of extinctions, sea level changes, and isotope variations. Palaeogeogr Palaeoclimatol Palaeoecol. 448: 224–244.

[16] Zong P , Ma X, Xue J, Jin X. 2016. Comparative study of Late Devonian (Famennian) brachiopod assemblages, sea level changes, and geo-events in northwestern and southern China. Palaeogeogr Palaeoclimatol Palaeoecol. 448: 298–316.

[17] Vleeschouwer D et al. 2017. Timing and pacing of the Late Devonian mass extinction event regulated by eccentricity and obliquity. Nat Commun. 8: 2268. 29273792 10.1038/s41467-017-02407-1 PMC5741662

[18] Joachimski MM , Buggisch W. 1993. Anoxic events in the late Frasnian—causes of the Frasnian–Famennian faunal crisis. Geology. 21: 675–678.

[19] Turgeon SC , Creaser RA, Algeo TJ. 2007. Re–Os depositional ages and seawater Os estimates for the Frasnian–Famennian boundary: implications for weathering rates, land plant evolution, and extinction mechanism. Earth Planet Sci Lett. 261: 649–661.

[20] Percival LME , et al. 2018. Precisely dating the Frasnian-Famennian boundary: Implications for the cause of the Late Devonian mass extinction. Sci Rep. 8: 9578. 29934550 10.1038/s41598-018-27847-7 PMC6014997

[21] Becker RT , Gradstein FM, Hammer O. 2012. The Devonian period. In: Gradstein FM, Ogg JG, Schmitz M, Ogg G, editors. Geological time scale 2012, vol. 2. Amsterdam: Elsevier. p. 559–601.

[22] Chen DZ , Tucker ME. 2003. The Frasnian–Famennian mass extinction: insights from high-resolution sequence stratigraphy and cyclostratigraphy in South China. Palaeogeogr Palaeoclimatol Palaeoecol. 193: 87–111.

[23] Huang C , Gong YM. 2016. Timing and patterns of the Frasnian–Famennian event: evidences from high-resolution conodont biostratigraphy and event stratigra-phy at the Yangdi section, Guangxi, South China. Palaeogeogr Palaeoclimatol Palaeoecol. 448: 317–338.

[24] Clarkson MO et al. 2014. Assessing the utility of Fe/Al and Fe-speciation to record water column redox conditions in carbonate-rich sediments. Chem Geol. 382: 111–122.

[25] Raiswell R et al. 2008. Turbidite depositional influences on the diagenesis of Beecher’s Trilobite Bed and the Hunsruck Slate; sites of soft tissue pyritization. Am J Sci. 308: 105–129.

[26] Poulton SW , Canfield DE. 2011. Ferruginous conditions: a dominant feature of the ocean through Earth’s history. Elements. 7: 107–112.

[27] Joachimski MM et al. 2001. Water column anoxia, enhanced productivity and concomitant changes in δ^13^C and δ^34^S across the Frasnian–Famennian boundary (Kowala—Holy Cross Mountains/Poland). Chem Geol. 175: 109–131.

[28] John EH , Wignall PB, Newton RJ, S. H. 2010. Bottrell, δ^34^S_CAS_ and δ^18^O_CAS_ records during the Frasnian–Famennian (Late Devonian) transition and their bearing on mass extinction models. Chem Geol. 275: 221–234.

[29] Marenco PJ , Corsetti FA, Hammond DE, Kaufman AJ, Bottjer DJ. 2008. Oxidation of pyrite during extraction of carbonate associated sulfate. Chem Geol. 247: 124–132.

[30] Chen DZ et al. 2013. Large sulphur isotopic perturbations and oceanic changes during the Frasnian–Famennian transition of the Late Devonian. J Geol Soc London. 170: 465–476.

[31] Peng Y et al. 2014. Widespread contamination of carbonate-associated sulfate by present-day secondary atmospheric sulfate: evidence from triple oxygen isotopes. Geology. 42: 815–818.

[32] Wotte T , Shields-Zhou GA, Strauss H. 2012. Carbonate-associated sulfate: experimental comparisons of common extraction methods and recommendations toward a standard analytical protocol. Chem Geol. 326–327: 132–144.

[33] Strauss H , Marais DJD, Hayes JM, Summons RE. 1992. Concentrations of organic carbon and maturities and elemental compositions of kerogens. In: Schopf WJ, Klein C, editors. The Proterozoic biosphere: a multidisciplinary study. New York (NY): Cambridge University Press. p. 95–99.

[34] Amrani A , Lewan MD, Aizenshtat Z. 2005. Stable sulfur isotope partitioning during simulated petroleum formation as determined by hydrous pyrolysis of Ghareb Limestone, Israel. Geochim Cosmochim Acta. 69: 5317–5331.

[35] Cai CF et al. 2009. Distinguishing Cambrian from Upper Ordovician source rocks: evidence from sulfur isotopes and biomarkers in the Tarim Basin. Org Geochem. 40: 755–768.

[36] Li C et al. 2012. Evidence for a redox stratified cryogenian marine basin, datangpo formation, South China. Earth Planet Sci Lett. 331–332: 246–256.

[37] Yuan YY , et al. 2014. Redox condition during Ediacaran–Cambrian transition in the Lower Yangtze deep water basin, South China: constraints from iron speciation and δ^13^C_org_ in the Diben section, Zhejiang. Chin Sci Bull. 59: 3638–3649.

[38] Chen DZ , Qing HR, Li RW. 2005. The Late Devonian Frasnian–Famennian (F/F) biotic crisis: insights from δ^13^C_carb_, δ^13^C_org_, ^87^Sr/^86^Sr isotopic systematics. Earth Planet Sci Lett. 235: 151–166.

[39] Wignall P , Newton R. 1998. Pyrite framboid diameter as a measure of oxygen deficiency in ancient mudrocks. Am J Sci. 298: 537–552.

[40] Zhao H . 2022. Mercury isotope evidence for regional volcanism during the Frasnian–Famennian transition. Earth Planet Sci Lett. 581: 117412.

[41] Raven MR , Sessions AL, Fischer WW, Adkins JF. 2016. Sedimentary pyrite δ^34^S differs from porewater sulfide in Santa Barbara Basin: proposed role of organic sulfur. Geochim Cosmochim Acta. 186: 120–134.

[42] Pkovalevich VM , Peryt TM, Petrichenko OI. 1998. Secular variation in seawater chemistry during the Phanerozoic as indicated by brine inclusions in halite. J Geol. 106: 695–712.

[43] Makhnach A , Shimanovich V, Streltsova G, Mikhaylov N. 2014. Anhydrite and gypsum in the Devonian and Permian evaporite lithofacies of Belarus: a review. Geol Q. 58: 577–590.

[44] Machel HG . 2013. Secondary anhydrites in deeply buried Devonian carbonates of the Alberta Basin, Canada. Carbonates Evaporites. 28: 267–280.

[45] Peryt TM et al. 2007. Sulfur isotopes in anhydrites from the Upper Devonian Prypiac' and Dnipro-Donets Basins (Belarus and Ukraine). Carbonates Evaporites. 22: 43–54.

[46] Menor-Salván C , Tornos F, Fernández-Remolar D, Amils R. 2010. Association between catastrophic paleovegetation changes during Devonian–Carboniferous boundary and the formation of giant massive sulfide deposits. Earth Planet Sci Lett. 299: 398–408.

[47] Canfield DE , Farquhar J, Zerkle AL. 2010. High isotope fractionations during sulfate reduction in a low-sulfate euxinic ocean analog. Geology. 38: 415–418.

[48] Habicht KS , Gade M, Thamdrup B, Berg P, Canfield DE. 2002. Calibration of sulfate levels in the Archean Ocean. Science. 298: 2372–2374. 12493910 10.1126/science.1078265

[49] Ries JB , Fike DA, Pratt LM, Lyons TW, Grotzinger JP. 2009. Superheavy pyrite (δ^34^S_pyr_ >δ^34^S_CAS_) in the terminal Proterozoic Nama Group, southern Nambia; a consequence of low seawater sulfate at the dawn of animal life. Geology. 37: 743–746.

[50] Johnson JG , Klapper G, Sandberg CA. 1985. Devonian eustatic fluctuations in Euramerica. Geol Soc Am Bull. 96: 567–587.

[51] Hallam A , Wignall PB. 1999. Mass extinctions and sea-level changes. Earth Sci Rev. 48: 217–250.

[52] Gomes ML , Johnston DT. 2017. Oxygen and sulfur isotopes in sulfate in modern euxinic systems with implications for evaluating the extent of euxinia in ancient oceans. Geochim Cosmochim Acta. 205: 331–359.

[53] Fakhraee M , Li JY, Katsev S. 2017. Significant role of organic sulfur in supporting sedimentary sulfate reduction in low-sulfate environments. Geochim Cosmochim Acta. 213: 516–502.

[54] Sageman BB et al. 2003. A tale of shales: the relative roles of production, decomposition, and dilution in the accumulation of organic-rich strata, Middle–Upper Devonian, Appalachian basin. Chem Geol. 195: 229–273.

[55] Percival LM et al. 2020. Phosphorus-cycle disturbances during the Late Devonian anoxic events. Glob Planet Change. 184: 103070.

[56] Moreno C , Gonzalez F, Sáez R, Melgarejo JC, Suárez-Ruiz I. 2018. The Upper Devonian Kellwasser event recorded in a regressive sequence from inner shelf to lagoonal pond, Catalan Coastal Ranges, Spain. Sedimentology. 65: 2055–2087.

[57] Laakso TA , Schrag DP. 2019. A small marine biosphere in the Proterozoic. Geobiology. 17: 161–171. 30417524 10.1111/gbi.12323

[58] Reinhard CT et al. 2017. Evolution of the global phosphorus cycle. Nature. 541: 386–389. 28002400 10.1038/nature20772

[59] Cai CF et al. 2009. Distinguishing Cambrian from Upper Ordovician source rocks: evidence from sulfur isotopes and biomarkers in the Tarim Basin. Org Geochem. 40: 755–768.

[60] Clarkson MO , et al. 2016. Dynamic anoxic ferruginous conditions during the end-Permian mass extinction and recovery. Nat Commun. 7: 1–9. 10.1038/ncomms12236 PMC4960316 27433855

[61] Halevy I , Peters SE, Fischer WW. 2012. Sulfate burial constraints on the Phanerozoic sulfur cycle. Science. 337: 331–334. 22822147 10.1126/science.1220224

[62] Kipp MA , Tissot FLH. 2022. Inverse methods for consistent quantification of seafloor anoxia using uranium isotope data from marine sediments. Earth Planet Sci Lett. 577: 117240.

[63] Ingvorsen K , Jørgensen BB. 1984. Kinetics of sulfate uptake by freshwater and marine species of Desulfovibrio. Arch Microbiol. 139: 61–66.

[64] Canfield DE . 1989. Sulfate reduction and oxic respiration in marine sediments: implications for organic carbon preservation in euxinic environments. Deep Sea Res A. 36: 121–138. 11542177 10.1016/0198-0149(89)90022-8

[65] Lee J , et al. 2019. Natural and anthropogenic signatures on sedimentary organic matters across varying intertidal habitats in the Korean waters. Environ Int. 133: 105166. 31518928 10.1016/j.envint.2019.105166

[66] Li XX , Zhang ZR, Wade TL, Knap AH, Zhang CL. 2017. Sources and compositional distribution of organic carbon in surface sediments from the lower Pearl River to the coastal South China Sea. J Geophys Res Biogeosci. 122: 2104–2117.

[67] Urban NR , Brezonik PL, Baker LA, Sherman LA. 1994. Sulfate reduction and diffusion in sediments of Little Rock Lake, Wisconsin. Limnol Oceanogr. 39: 797–815.

