# Supplementary material for: Significant fluctuation in the global sulfate reservoir and oceanic redox state during the Late Devonian event
Source: PNAS Nexus. 2022 Jul 30;1(4):pgac122. doi: 10.1093/pnasnexus/pgac122 (PMC9802379; doi:10.1093/pnasnexus/pgac122)
Supplement: pgac122_Supplemental_File [file pgac122_supplemental_file.docx]

SUPPLEMENTARY INFORMATION

Table of Contents

[1 Sampling, Regional Sea Level Change, Biomass Distribution and Stratigraphic Correlation 1](#_Toc36139609)

[*1.1 Sampling* 1](#_Toc36139610)

[*1.2* Regional Sea Level Change, Biomass Distribution and Stratigraphic Correlation 1](#_Toc36139611)

[2 Calculation of Contamination from Pyrite S in Kerogen after the Treatments 2](#_Toc36139612)

[References 3](#_Toc36139626)

[Supplementary Tables 5](#_Toc36139627)

Supplementary [Figures 8](#_Toc36139628)

# 1 Sampling, Regional Sea Level Change, Biomass Distribution and Stratigraphic Correlation

## *1.1 Sampling*

A total of thirty three rock samples at Frasnian-Famennian boundary (FFB) were collected from well-exposed Xikuangshan section, which is located at 27°45'56''N and 111°29'51''E, ~12 km north of Lengshuijiang City in Hunan Province, South China (Fig. S1). These samples were selected without considering TOC and lithology, but less weathered samples were preferentially chosen. Weathered surfaces and veins were carefully removed during sampling during field work and subsequently in laboratory.

## *1.2 Regional Sea Level Change, Biomass Distribution and Stratigraphic Correlation*

The FFB sequence in the studied area shows large scale regression from the Upper *Pa. rhenana* zone (below the base of the studied section) to the uppermost portion of the *linguiformis* zone in the latest Frasnian. This regressive sequence is followed by a transgression that starts at the base of the Middle *triangularis* zone, and that overlies the undulating and current scouring structures found in the Lower *triangularis* zone*.* The transgressive sequence is characterized by well-laminated mudstones and shales (upward from the bottom of L10) (1,2) (Fig. 1). Frequent sea-level fluctuations are superimposed on these longer-term regressive and transgressive events (1-4) (Fig. 1). Importantly, though the F–F black shale horizon (L6) represents a sea-level rise event in terms of lithologic change, this interval was still deposited in a shallow-water environment^1^. This scenario is consistent with some other sections in South China (4-6), which indicate that sea-level was rising at a small-scale, but falling in a greater third-order sequence on a longer timescale (ref. 6; Fig. 1). The organic matter rich UKH, found just below F-F boundary in the uppermost *linguiformis* zone, is the main extinction pulse of the prolonged, stepwise collapse of the Devonian ecosystem (4,7,8). Quantitative fossil records constrained by conodont biostratigraphy reveal that two steps of the Upper Kellwasser Crisis are registered at the base and near the top of the UKH in South China, respectively (2,4). The number and diversity of ostracods, corals, and brachiopods were very high near the top of the Frasnian. In contrast, the most signifcant biomass and diversity loss occurred at the UKH in the base of the black shale (L6) (2,3), which is similar to some localities in Europe (9,10). After this interval, corals disappeared completely, most of brachiopods vanished except for rare *Radiatrypa maanshanensis* and *Desquamatia shetienchiaoensis,* which remain in Famennian; but ostracods remained abundant although the diversity is very low. Thus, some bryozoans, brachiopods, and rare ostracods can be found in the black-shale interval (1,2,3). The second step of the UKH mass extinction occurred in the FFB with dramatic reduction of the ostracod and further elimination of brachiopods.

The positive excursions in δ^13^C_org_ or δ^13^C_carb_ in the upmost *linguiformis* zone have been reported globally. The amplitude of this positive excursion at Xikuangshan is ~2‰ in this study (Fig. 1), which is comparable to the UKH excursions in δ^13^C_carb_ in USA (~3‰; ref. 11), Germany (~3‰; ref. 12), Poland (~3‰; ref. 13-14), and other South China profiles (~2.5‰ to 4‰; ref. 15-17). The stratigraphic classification in our section is generally in accordance with Ma et al. (ref. 2; Fig. 1, L4–L10), in which the UKH is generally continuous, but the LKH has no exposed counterpart outcrop in the study area.

# 2 Calculation of Contamination from Pyrite S in Kerogen after the Treatments

Based on the data mentioned above, organic sulfur content in the kerogen (S_Kero_%) was subsequently calculated by subtraction of the S_Py_ from the total residual kerogen sulfur as following steps:

The mass of iron in the dissolved sulfate solution (M_Fe_) can be expressed by equation:

M_Fe_=C_Fe_×200×10^-6^. (DR1)

where C_Fe_ is concentration of iron, in mg/L.

Then the mass of residual pyrite in the extracted kerogen (M_Py_) is:

M_Py_=120×M_Fe_/56. (DR2)

So the proportion of residual pyrite in the extracted kerogen is:

Py%=(M_FePy_/M_k_)×100%. (DR3)

For the proportion of S_Py_ in the extracted kerogen:

S_Py_%= Py%×64/120. (DR4)

The total residual kerogen S can be calculated as the S in BaSO_4_ (S_BaSO4_%):

S_BaSO4_%=(32×M_BaSO4_)/(233×M_Kero_). (DR5)

Finally, S_Kero_% can be expressed as:

S_Kero_%=(S_BaSO4_%- S_FePy_%)/S_BaSO4_%. (DR6)

We now substitute Equation DR1~DR5 into Equation DR6 and obtain a S_Kero_% value. When S_Kero_% ≥92%, an error due to contamination from ≤8% S_Py_ is estimated to be <0.8‰ given the differences in δ^34^S between kerogen and pyrite generally <10‰ (refs. 28,29). If not, more treatments using HCl and CrCl_2_ should be employed.

The S_Kero_% values of the 15 residual kerogen samples after acidic chromous chloride treatments range from 92.57–99.46%, suggesting that the pyrite in all samples have been well removed (Table S1). Thus, the residual kerogen samples can be used for the following analyses of δ^34^S_Kero_.

# Supplementary references

1 Ma, X.P. & Bai, S.L. Biological, depositional, microspherule, and geochemical records of the Frasnian/Famennian boundary beds, South China. *Palaeogeography Palaeoclimatology Palaeoecology* **181**, 325–346 (2002).

2 Ma, X.P., Gong, Y., Chen, D. & Racki, G. The Late Devonian Frasnian–Famennian event in South China—Patterns and causes of extinctions, sea level changes, and isotope variations. *Palaeogeography Palaeoclimatology Palaeoecology* **448**, 224–244 (2016).

3 Zong, P., Ma, X., Xue, J. & Jin, X. Comparative study of Late Devonian (Famennian) brachiopod assemblages, sea level changes, and geo-events in northwestern and southern China. *Palaeogeography Palaeoclimatology Palaeoecology* **448**, 298–316 (2016).

4 Huang, C., [Joachimski, M.M](http://apps.webofknowledge.com/OutboundService.do?SID=7EGXbaRUhHynzk91KD3&mode=rrcAuthorRecordService&action=go&product=WOS&daisIds=217241). & [Gong, Y.M](http://apps.webofknowledge.com/OutboundService.do?SID=7EGXbaRUhHynzk91KD3&mode=rrcAuthorRecordService&action=go&product=WOS&daisIds=523374). Did climate changes trigger the Late Devonian Kellwasser Crisis? Evidence from a high-resolution conodont δ^18^O_PO4_ record from South China. Earth and Planetary Science Letters 495, 174–184 (2018).

5 Chen, D.Z., Tucker, M.E., Shen, Y., Yans, J. & Preat, A. Carbon isotope excursions and sea-level change, Implications for the Frasnian-Famennian biotic crisis. *Journal of the Geological Society (London)* **159**, 623–626 (2002).

6 Wang, X., [Liu, S.A](http://apps.webofknowledge.com/OutboundService.do?SID=7EGXbaRUhHynzk91KD3&mode=rrcAuthorRecordService&action=go&product=WOS&daisIds=891801)., [Wang, Z.R](http://apps.webofknowledge.com/OutboundService.do?SID=7EGXbaRUhHynzk91KD3&mode=rrcAuthorRecordService&action=go&product=WOS&daisIds=2116470)., [Chen, D.Z](http://apps.webofknowledge.com/OutboundService.do?SID=7EGXbaRUhHynzk91KD3&mode=rrcAuthorRecordService&action=go&product=WOS&daisIds=802762). & [Zhang, L.Y](http://apps.webofknowledge.com/OutboundService.do?SID=7EGXbaRUhHynzk91KD3&mode=rrcAuthorRecordService&action=go&product=WOS&daisIds=10422976). Zinc and strontium isotope evidence for climate cooling and constraints on the Frasnian–Famennian (~372Ma) mass extinction. *Palaeogeography Palaeoclimatology Palaeoecology* **498**, 68–82 (2018).

7 Vleeschouwer, D., [Da Silva, A.C](http://apps.webofknowledge.com/OutboundService.do?SID=7EGXbaRUhHynzk91KD3&mode=rrcAuthorRecordService&action=go&product=WOS&daisIds=935442)., [Sinnesael, M](http://apps.webofknowledge.com/OutboundService.do?SID=7EGXbaRUhHynzk91KD3&mode=rrcAuthorRecordService&action=go&product=WOS&daisIds=7642212)., [Chen, D.Z](http://apps.webofknowledge.com/OutboundService.do?SID=7EGXbaRUhHynzk91KD3&mode=rrcAuthorRecordService&action=go&product=WOS&daisIds=802762). , [Day, J.E](http://apps.webofknowledge.com/OutboundService.do?SID=7EGXbaRUhHynzk91KD3&mode=rrcAuthorRecordService&action=go&product=WOS&daisIds=2374300)., [Whalen, M.T](http://apps.webofknowledge.com/OutboundService.do?SID=7EGXbaRUhHynzk91KD3&mode=rrcAuthorRecordService&action=go&product=WOS&daisIds=8533539)., [Guo, Z.H](http://apps.webofknowledge.com/OutboundService.do?SID=7EGXbaRUhHynzk91KD3&mode=rrcAuthorRecordService&action=go&product=WOS&daisIds=4814270). & [Claeys, P](http://apps.webofknowledge.com/OutboundService.do?SID=7EGXbaRUhHynzk91KD3&mode=rrcAuthorRecordService&action=go&product=WOS&daisIds=178200). Timing and pacing of the Late Devonian mass extinction event regulated by eccentricity and obliquity. *Nature Communications* **8**, 2268 (2017).

8 McGhee, G.R.When the Invasion of Land Failed: The Legacy of the Devonian Extinctions.*Columbia University Press, New York, USA,* 336 (2013).

9 Joachimski, M.M. & Buggisch, W. Anoxic events in the late Frasnian—Causes of the Frasnian–Famennian faunal crisis. *Geology* **21**, 675–678 (1993).

10 Turgeon, S.C., Creaser, R.A. & Algeo, T.J. Re–Os depositional ages and seawater Os estimates for the Frasnian–Famennian boundary: Implications for weathering rates, land plant evolution, and extinction mechanism. *Earth and Planetary Science Letters* **261**, 649–661(2007).

11 Sim, M.S., Ono, S. & Hurtgen, M.T. Sulfur isotope evidence for low and fluctuating sulfate levels in the late Devonian ocean and the potential link with the mass extinction event. *Earth and Planetary Science Letters* **419**, 52–62 (2015).

12 Joachimski, M.M. & Buggisch, W. Conodont apatite δ^18^O signatures indicate climatic cooling as a trigger of the Late Devonian mass extinction. *Geology* **30**, 711–714 (2002).

13 Joachimski, M.M., [Ostertag-Henning, C](http://apps.webofknowledge.com/OutboundService.do?SID=7EGXbaRUhHynzk91KD3&mode=rrcAuthorRecordService&action=go&product=WOS&daisIds=1485310)., [Pancost, R.D](http://apps.webofknowledge.com/OutboundService.do?SID=7EGXbaRUhHynzk91KD3&mode=rrcAuthorRecordService&action=go&product=WOS&daisIds=75271)., [Strauss, H](http://apps.webofknowledge.com/OutboundService.do?SID=7EGXbaRUhHynzk91KD3&mode=rrcAuthorRecordService&action=go&product=WOS&daisIds=176734)., [Freeman, K.H](http://apps.webofknowledge.com/OutboundService.do?SID=7EGXbaRUhHynzk91KD3&mode=rrcAuthorRecordService&action=go&product=WOS&daisIds=134490)., [Littke, R](http://apps.webofknowledge.com/OutboundService.do?SID=7EGXbaRUhHynzk91KD3&mode=rrcAuthorRecordService&action=go&product=WOS&daisIds=83875)., [Damste, J.S.S](http://apps.webofknowledge.com/OutboundService.do?SID=7EGXbaRUhHynzk91KD3&mode=rrcAuthorRecordService&action=go&product=WOS&daisIds=5079). & [Racki, G](http://apps.webofknowledge.com/OutboundService.do?SID=7EGXbaRUhHynzk91KD3&mode=rrcAuthorRecordService&action=go&product=WOS&daisIds=480711). Water column anoxia, enhanced productivity and concomitant changes in δ13C and δ34S across the Frasnian–Famennian boundary (Kowala — Holy Cross Mountains/ Poland). *Chemical Geology* **175**, 109–131 (2001).

14 Kaiho, K., [Yatsu, S](http://apps.webofknowledge.com/OutboundService.do?SID=8AvYekWDShWrnXe9Rvl&mode=rrcAuthorRecordService&action=go&product=WOS&daisIds=10970171)., [Oba, M](http://apps.webofknowledge.com/OutboundService.do?SID=8AvYekWDShWrnXe9Rvl&mode=rrcAuthorRecordService&action=go&product=WOS&daisIds=1361243)., [Gorjan, P](http://apps.webofknowledge.com/OutboundService.do?SID=8AvYekWDShWrnXe9Rvl&mode=rrcAuthorRecordService&action=go&product=WOS&daisIds=3574403)., [Casier, J.G](http://apps.webofknowledge.com/OutboundService.do?SID=8AvYekWDShWrnXe9Rvl&mode=rrcAuthorRecordService&action=go&product=WOS&daisIds=1595190). & [Ikeda, M](http://apps.webofknowledge.com/OutboundService.do?SID=8AvYekWDShWrnXe9Rvl&mode=rrcAuthorRecordService&action=go&product=WOS&daisIds=3585373). A forest fire and soil erosion event during the Late Devonian mass extinction. *Palaeogeogr. Palaeoclimatol. Palaeoecol* **392**, 272–280 (2013).

15 Chen, D.Z., Qing, H.R. & Li, R.W. The Late Devonian Frasnian–Famennian (F/F) biotic crisis: Insights from δ^13^C_carb_, δ^13^C_org_, ^87^Sr/^86^Sr isotopic systematics. *Earth and Planetary Science Letters* **235**, 151–166 (2005).

16 Xu, B., Gu, Z., Han J & Wang, C. Environmental changes during Frasnian-Famennian transition in south China: A multiproxy approach. *Journal of Geophysical Research* **113**, 1754–1755 (2008).

17 Xu, B., [Gu, Z.Y](http://apps.webofknowledge.com/OutboundService.do?SID=7EGXbaRUhHynzk91KD3&mode=rrcAuthorRecordService&action=go&product=WOS&daisIds=801326)., [Wang, C.Y](http://apps.webofknowledge.com/OutboundService.do?SID=7EGXbaRUhHynzk91KD3&mode=rrcAuthorRecordService&action=go&product=WOS&daisIds=30315185)., [Hao, Q.Z](http://apps.webofknowledge.com/OutboundService.do?SID=7EGXbaRUhHynzk91KD3&mode=rrcAuthorRecordService&action=go&product=WOS&daisIds=757586)., [Han, J.T](http://apps.webofknowledge.com/OutboundService.do?SID=7EGXbaRUhHynzk91KD3&mode=rrcAuthorRecordService&action=go&product=WOS&daisIds=1157632)., [Liu, Q](http://apps.webofknowledge.com/OutboundService.do?SID=7EGXbaRUhHynzk91KD3&mode=rrcAuthorRecordService&action=go&product=WOS&daisIds=88581)., [Wang, L](http://apps.webofknowledge.com/OutboundService.do?SID=7EGXbaRUhHynzk91KD3&mode=rrcAuthorRecordService&action=go&product=WOS&daisIds=1509486). & [Lu, Y.W](http://apps.webofknowledge.com/OutboundService.do?SID=7EGXbaRUhHynzk91KD3&mode=rrcAuthorRecordService&action=go&product=WOS&daisIds=909993). Carbon isotopic evidence for the associations of decreasing atmospheric CO_2_ level with the Frasnian–Famennian mass extinction. *Journal of Geophysical Research* **117**, 65–65 (2012).

# Supplementary Tables

**Table S1 Phosphorus contents and TOC/P ratios in FFB sediments from worldwide basins**

| **Xikuangshan (China)** | | | | | **Appalachian basin (USA)*** | | | | | | | **Coumiac (France)**** | | | | | | | | | | | | **Steinbruch Schmidt (Germany)**** | | | | | | | | | | | | | | | | | | |  |
| --- | --- | --- | --- | --- | --- | --- | --- | --- | --- | --- | --- | --- | --- | --- | --- | --- | --- | --- | --- | --- | --- | --- | --- | --- | --- | --- | --- | --- | --- | --- | --- | --- | --- | --- | --- | --- | --- | --- | --- | --- | --- | --- | --- |
| **Conodont zone** | **Hei-ght** | **Age** | **P** | **TOC/P** | **Hei-ght** | **Conodont zone** | **Original depth** | | **P** | | **TOC/P** | | **Height** | | **Conodont zone** | | **Sam-**  **ple** | | **Original height** | | **P** | | **TOC/P** | | **Hei-ght** | | **Conodont zone** | | | **Sam-**  **ple** | | **Height** | | | | **P** | | **TOC**  **/P** | | |  |  |  |
|  | **(m)** | **(Ma)** | **(ppm)** |  | **(m)** |  | **(m)** | **(ppm)** | |  | | **(m)** | |  | |  | | **(m)** | | **(ppm)** | |  | | **(m)** | |  | |  | | | **(m)** | | | **(ppm)** | | |  | | |  |  |  |  |
| L *triang* | 5.47 | 371.94 | 149 | 10.1 |  |  |  |  | |  | |  | |  | |  | |  | |  | |  | | 5.85 | | L triangle | | | SCH 102 | | | | 3.82 | |  | | | |  | | |  |  |
| L *triang* | 5.21 | 371.96 | 114 | 21.4 |  |  |  |  | |  | | 6.74 | | M triang | | CM36 | | 14.80 | | 453 | | 2.3 | |  | | L triangle | | | SCH 101 | | | | 3.67 | |  | | | |  | | |  |  |
| L *triang* | 4.88 | 371.97 | 451 | 1.2 | 5.00 | L triangle | 235.16 | 114 | | 4.7 | | 6.25 | | L triangle | | CM35 | | 13.50 | | 410 | | 6.3 | |  | | L triangle | | | SCH 100 | | | | 3.49 | |  | | | |  | | |  |  |
| L *triang* | 4.64 | 371.99 | 268 | 1.9 | 4.70 | L triangle | 235.55 | 93 | | 26.2 | | 5.70 | | L triangle | | CM34 | | 12.20 | | 215 | | 8.4 | | 4.56 | | L triangle | | | SCH U7 | | | | 3.35 | | 1022 | | | | 27.3 | | |  |  |
| FFB | 4.42 | 372.00 |  |  |  |  |  |  | |  | |  | |  | |  | |  | |  | |  | |  | |  | | |  | | | |  | |  | | | |  | | |  |  |
|  | 4.40 | 372.01 | 175 | 24.5 | 4.40 |  | 236.00 | 89 | | 4.9 | | 4.50 | |  | | CM32a | | 9.20 | | 200 | | 3.9 | | 4.40 | |  | | | SCH U6 | | | | 3.27 | | 476 | | | | 29.2 | | |  |  |
|  | 4.19 | 372.06 | 247 | 9.1 | 4.19 |  | 236.35 | 96 | | 53.1 | | 4.40 | |  | | CM31g | | 9.10 | | 223 | | 33.6 | | 4.01 | |  | | | SCH U5 | | | | 3.16 | | 1330 | | | | 21.3 | | |  |  |
|  | 4.07 | 372.09 | 195 | 44.8 | 3.60 |  | 236.65 | 124 | | 48.4 | |  | |  | |  | |  | |  | |  | | 3.73 | |  | | | SCH U4 | | | | 3.08 | | 284 | | | | 39.0 | | |  |  |
|  | 3.85 | 372.14 | 400 | 22.8 |  |  |  |  | |  | |  | |  | |  | |  | |  | |  | | 3.59 | |  | | | SCH U3 | | | | 3.02 | | 930 | | | | 81.3 | | |  |  |
| Start of UKH | 3.59 | 372.20 |  |  |  |  |  |  | |  | |  | |  | |  | |  | |  | |  | |  | |  | | |  | | | |  | |  | | | |  | | |  |  |
| Pre-UKH/*linguiformis* | 3.42 | 372.21 | 471 | 13.4 | 3.40 | Pre-UKH/*linguiformis* | 236.98 | 84 | | 77.4 | | 3.30 | | Pre-  UKH/ *linguiformis* | | CM31f | | 9.00 | |  | |  | | 3.48 | | Pre-  UKH/ *linguiformis* | | | SCH U3 BASE | | | | 2.97 | | 1050 | | | | 19.4 | | |  |  |
|  | 3.21 | 372.22 | 299 | 7.2 | 3.25 |  | 237.28 | 78 | | 69.2 | | 3.00 | |  | | CM31c | | 8.70 | | 426 | | 3.6 | | 3.40 | |  | | | SCH U1 | | | | 2.94 | | 955 | | | | 18.9 | | |  |  |
|  | 2.97 | 372.23 | 275 | 4.8 | 3.06 |  | 237.64 | 87 | | 3.0 | | 2.50 | |  | | CM30 | | 8.25 | |  | |  | | 3.35 | |  | | | SCH 56 | | | | 2.86 | | 374 | | | | 2.8 | | |  |  |
|  | 2.70 | 372.25 | 198 | 12.6 | 2.89 |  | 237.96 | 112 | | 1.6 | |  | |  | |  | |  | |  | |  | | 3.02 | |  | | | SCH 54 | | | | 2.64 | | 213 | | | | 3.6 | | |  |  |
|  | 2.49 | 372.26 | 317 | 6.9 | 2.73 |  | 238.26 | 122 | | 1.3 | |  | |  | |  | |  | |  | |  | | 2.30 | |  | | | SCH 51 | | | | 2.15 | | 244 | | | | 3.2 | | |  |  |
| Pre-UKH/*linguiformis* | 2.23 | 372.28 | 163 | 15.5 | 0.00 |  | 243.34 |  | |  | |  | |  | |  | |  | |  | |  | |  | |  | | |  | | | |  | |  | | | |  | | |  |  |
|  | 0 | 372.40 |  |  |  |  |  |  | |  | |  | |  | |  | |  | |  | |  | |  | |  | | |  | | | |  | |  | | | |  | | |  |  |

Note: 1) * from Sageman et al. (2003);** from Percival et al.(2020).

2) Original height or depth is corrected relative to Xikuanshan section according to conodont zones; green area represents UKH.

3) All P contents were measured using XRF.

**Table S2 Geochemical data from Xikuangshan section**

| Sample  number | Height  (m) | TOC (wt.%) | δ^13^C_org_ (‰) | δ^34^S_Kero_ (‰) | δ^34^S_CAS_ (‰) | δ^34^S_Py_ (‰) | △^34^S_CAS-Py_ (‰) | △^34^S_Kero-Py_ (‰) | Fe_T_ (%) | Fe_carb_ (%) | Fe_Ox_ (%) | Fe_Mag_ (%) | Fe_Py_ (%) | Fe_HR_ (%) | Fe_HR_/  Fe_T_ | Fe_Py_/  Fe_HR_ | Py  (wt.%) | S_Py_  (wt.%) | S_Kero_ (wt.%) | (H/C)_Kero (molar ratio)_ |
| --- | --- | --- | --- | --- | --- | --- | --- | --- | --- | --- | --- | --- | --- | --- | --- | --- | --- | --- | --- | --- |
| X5 | 0.19 | 0.25 | -26.95 | --** | -- | -- | -- | -- | 1.30 | 0.04 | 0.11 | 0.07 | 0.01 | 0.22 | 0.17 | 0.04 | 0.02 | 0.01 | -- | -- |
| X6 | 0.37 | 0.08 | -- | -- | -- | -- | -- | -- | 0.38 | 0.00 | 0.00 | 0.00 | -- | 0.01 | 0.02 | -- | -- | -- | -- | -- |
| X7 | 0.55 | 0.09 | -26.34 | -- | -- | -- | -- | -- | 0.37 | 0.00 | 0.00 | 0.00 | -- | 0.01 | 0.02 | -- | -- | -- | -- | -- |
| X8 | 0.94 | 0.16 | -26.20 | -- | -- | -- | -- | -- | 2.21 | 0.01 | 0.01 | 0.03 | -- | 0.04 | 0.02 | -- | -- | -- | -- | -- |
| X9 | 1.23 | 0.15 | -26.79 | -- | -- | -- | -- | -- | 2.47 | 0.11 | 0.38 | 0.13 | 0.06 | 0.68 | 0.28 | 0.08 | 0.12 | 0.06 | -- | -- |
| X10 | 1.51 | 0.34 | -- | -- | -- | -- | -- | -- | 2.81 | 0.21 | 0.25 | 0.07 | -- | 0.53 | 0.19 | -- | -- | -- | -- | -- |
| X11 | 1.77 | 0.16 | -26.44 | -- | -- | -- | -- | -- | 3.38 | 0.20 | 0.16 | 0.39 | 0.03 | 0.77 | 0.23 | 0.04 | 0.07 | 0.04 | -- | -- |
| X12 | 1.98 | 0.24 | -26.22 | -- | -- | -- | -- | -- | 0.44 | 0.00 | 0.00 | 0.00 | -- | 0.01 | 0.02 | -- | -- | -- | -- | -- |
| X13 | 2.22 | 0.25 | -26.47 | 1.67 | 26.17 | -10.11 | 36.29 | 11.78 | 2.68 | 0.05 | 0.01 | 0.01 | 0.01 | 0.08 | 0.03 | 0.07 | 0.01 | 0.01 | 95.43 | 0.39 |
| X14 | 2.48 | 0.22 | -26.91 | -- | -- | -- | -- | -- | 2.23 | 0.17 | 0.05 | 0.11 | 0.05 | 0.38 | 0.17 | 0.12 | 0.10 | 0.05 | -- | -- |
| X15 | 2.69 | 0.25 | -26.71 | 0.33 | 21.26 | -8.58 | 29.85 | 8.92 | 2.13 | 0.01 | 0.24 | 0.03 | 0.13 | 0.41 | 0.19 | 0.33 | 0.29 | 0.15 | 97.53 | 0.67 |
| X16 | 2.96 | 0.13 | -26.84 | 9.81 | 22.86 | -2.73 | 25.59 | 12.53 | 2.75 | 0.08 | 0.36 | 0.05 | 0.20 | 0.69 | 0.25 | 0.29 | 0.43 | 0.23 | 96.89 | 0.54 |
| X17 | 3.20 | 0.34 | -26.65 | 15.43 | 23.47 | -1.43 | 24.91 | 16.87 | 0.79 | 0.11 | 0.04 | 0.00 | 0.07 | 0.23 | 0.29 | 0.33 | 0.16 | 0.09 | 92.98 | 0.41 |
| X18 | 3.41 | 0.40 | -26.71 | 15.66 | 26.22 | 2.32 | 23.91 | 13.35 | 0.54 | 0.01 | 0.04 | 0.01 | 0.00 | 0.06 | 0.11 | 0.03 | 0.00 | 0.00 | 98.70 | 0.76 |
| X19 | 3.58 | 0.87 | -26.22 | -9.33 | -- | -11.34 | -- | 2.01 | 3.87 | 0.04 | 0.51 | 0.03 | 2.82 | 3.40 | 0.88 | 0.83 | 6.05 | 3.23 | 95.97 | 1.37 |
| X20 | 3.84 | 0.91 | -25.15 | -7.30 | -- | -15.22 | -- | 7.93 | 4.62 | 0.02 | 1.43 | 0.07 | 1.86 | 3.38 | 0.73 | 0.55 | 3.98 | 2.12 | 98.16 | 0.40 |
| X21 | 4.06 | 0.87 | -26.03 | 2.96 | 14.97 | -10.06 | 25.04 | 13.02 | 5.68 | 0.16 | 0.95 | 0.04 | 1.58 | 2.72 | 0.48 | 0.58 | 3.39 | 1.81 | 99.46 | 0.66 |
| X22 | 4.18 | 0.23 | -25.81 | 4.08 | 7.07 | -6.76 | 13.83 | 10.84 | 6.18 | 2.84 | 0.11 | 0.09 | 0.21 | 3.24 | 0.53 | 0.06 | 0.44 | 0.24 | 96.76 | 0.56 |
| X23 | 4.39 | 0.43 | -26.14 | 3.62 | -- | -10.36 | -- | 13.98 | 3.49 | 0.04 | 1.11 | 0.01 | 0.38 | 1.54 | 0.44 | 0.25 | 0.82 | 0.44 | 92.87 | 0.49 |
| X24 | 4.63 | 0.05 | -25.60 | -- | 24.30 | 21.99 | 2.31 | -- | 4.77 | 0.01 | 0.66 | 1.54 | 0.27 | 2.48 | 0.52 | 0.11 | 0.58 | 0.31 | -- | -- |
| X25 | 4.87 | 0.06 | -25.66 | -- | 16.68 | 20.88 | -4.20 | -- | 1.46 | 0.07 | 0.22 | 0.02 | 0.02 | 0.34 | 0.23 | 0.07 | 0.05 | 0.03 | -- | -- |
| X26 | 5.20 | 0.24 | -25.97 | 11.16 | 24.39 | -0.61 | 25.01 | 11.78 | 4.31 | 0.13 | 0.91 | 0.00 | 0.29 | 1.34 | 0.31 | 0.22 | 0.63 | 0.34 | 97.17 | 0.42 |
| X27 | 5.46 | 0.15 | -25.61 | -- | 14.43 | 8.10 | 6.33 | -- | 2.29 | 0.04 | 0.02 | 0.02 | 0.01 | 0.09 | 0.04 | 0.09 | 0.02 | 0.01 | -- | -- |
| X28 | 5.74 | 0.25 | -25.36 | 8.23 | 21.54 | -9.67 | 31.21 | 17.90 | 2.96 | 0.17 | 0.53 | 0.01 | 0.03 | 0.74 | 0.25 | 0.04 | 0.06 | 0.03 | 98.94 | 0.48 |
| X29 | 5.98 | 0.25 | -25.76 | 9.23 | -- | -3.87 | -- | 13.10 | 3.52 | 0.05 | 0.18 | 0.01 | 0.04 | 0.28 | 0.08 | 0.13 | 0.08 | 0.04 | 95.72 | 0.65 |
| X30 | 6.27 | 0.37 | -- | -- | -- | -- | -- | -- | 1.83 | 0.10 | 0.19 | 0.01 | -- | 0.29 | 0.16 | -- | -- | -- | -- | -- |
| X31 | 6.63 | 0.28 | -25.55 | -- |  | 6.70 |  | -- | 4.50 | 0.03 | 0.40 | 0.01 | 0.15 | 0.58 | 0.13 | 0.25 | 0.31 | 0.17 | 92.57 | 1.65 |
| X32 | 7.04 | 0.90 | -- | -- | -- | -- | -- | -- | 3.36 | 0.12 | 0.63 | 0.00 | 0.46 | 1.21 | 0.36 | 0.38 | 0.98 | 0.52 |  |  |
| X33 | 7.60 | 0.55 | -25.83 | 8.52 |  | 1.11 |  | 7.42 | 2.71 | 0.43 | 0.55 | 0.00 | 0.46 | 1.43 | 0.53 | 0.32 | 0.98 | 0.52 | 98.35 | 1.77 |
| X34 | 8.26 | 1.44 | -- | -- | -- | -- | -- | -- | 3.02 | 0.12 | 0.60 | 0.00 | 0.70 | 1.42 | 0.47 | 0.49 | 1.49 | 0.79 | -- | -- |
| X35 | 8.77 | 0.90 | -25.77 | -- | -- | -- | -- | -- | 5.58 | 1.13 | 0.28 | 0.00 | 1.21 | 2.62 | 0.47 | 0.46 | 2.59 | 1.38 | -- | -- |
| X36 | 9.19 | 0.72 | -- | -- | -- | -- | -- | -- | 3.19 | 0.12 | 1.05 | 0.11 | 0.47 | 1.75 | 0.55 | 0.27 | 1.01 | 0.54 | -- | -- |
| X37 | 9.83 | 1.00 | -- | -- | -- | -- | -- | -- | 2.63 | 0.01 | 0.74 | 0.22 | 0.44 | 1.42 | 0.54 | 0.31 | 0.94 | 0.50 | -- | -- |

Note: * S_Kero_ is organic sulfur percentage in total organic matter after treatments, and **-- means not determined.

**Table S3** Values of areal fraction of euxinia, f1, and their range used in the stochastic analysis. The value of f1 for each sample is obtained by considering the maximum areal fraction of euxinia (0.2) and the maximum value of Fe_Py_/Fe_HR_. The range of f1 before the UKH where iron speciation results indicate oxic condition is assumed by considering the minimum anoxia in the modern ocean (0.5%) and the calculated f1 from the Fe_Py_/Fe_HR_ measurement. During the UKH, where the result from Fe_HR_/F_T_ indicate anoxic condition, the range of f1is obtained by considering the threshold between anoxic and oxic condition (Fe_Py_/ Fe_HR_  = 0.7), and the calculated f1 from the Fe_Py_/Fe_HR_ measurement.

| Sample  number | Height  (m) | TOC (%) | Fe_HR_/  Fe_T_ | Fe_Py_/  Fe_HR_ | f1 | Range f1 |
| --- | --- | --- | --- | --- | --- | --- |
| X5 | 0.19 | 0.25 | 0.17 | 0.04 | 0.01 | 0.005-0.01 |
| X6 | 0.37 | 0.08 | 0.02 | -- | -- | -- |
| X7 | 0.55 | 0.09 | 0.02 | -- | -- | -- |
| X8 | 0.94 | 0.16 | 0.02 | -- | -- | -- |
| X9 | 1.23 | 0.15 | 0.28 | 0.08 | 0.019 | 0.005-0.019 |
| X10 | 1.51 | 0.34 | 0.19 | -- | -- | -- |
| X11 | 1.77 | 0.16 | 0.23 | 0.04 | 0.01 | 0.005-0.019 |
| X12 | 1.98 | 0.24 | 0.02 | -- | -- | -- |
| X13 | 2.22 | 0.25 | 0.03 | 0.07 | 0.017 | 0.005-0.017 |
| X14 | 2.48 | 0.22 | 0.17 | 0.12 | 0.029 | 0.005-0.029 |
| X15 | 2.69 | 0.25 | 0.19 | 0.33 | 0.08 | 0.005-0.08 |
| X16 | 2.96 | 0.13 | 0.25 | 0.29 | 0.07 | 0.005-0.07 |
| X17 | 3.20 | 0.34 | 0.29 | 0.33 | 0.08 | 0.005-0.08 |
| X18 | 3.41 | 0.40 | 0.11 | 0.03 | 0.007 | 0.005-0.007 |
| X19 | 3.58 | 0.87 | 0.88 | 0.83 | 0.2 | 0.17-0.2 |
| X20 | 3.84 | 0.91 | 0.73 | 0.55 | 0.133 | 0.133-0.17 |
| X21 | 4.06 | 0.87 | 0.48 | 0.58 | 0.14 | 0.14-0.17 |
| X22 | 4.18 | 0.23 | 0.53 | 0.06 | 0.014 | 0.014-0.17 |
| X23 | 4.39 | 0.43 | 0.44 | 0.25 | 0.06 | 0.06-0.17 |
| X24 | 4.63 | 0.05 | 0.52 | 0.11 | 0.027 | 0.005-0.027 |
| X25 | 4.87 | 0.06 | 0.23 | 0.07 | 0.017 | 0.005-0.017 |
| X26 | 5.20 | 0.24 | 0.31 | 0.22 | 0.053 | 0.005-0.053 |
| X27 | 5.46 | 0.15 | 0.04 | 0.09 | 0.022 | 0.005-0.022 |
| X28 | 5.74 | 0.25 | 0.25 | 0.04 | 0.01 | 0.005-0.01 |
| X29 | 5.98 | 0.25 | 0.08 | 0.13 | 0.031 | 0.005-0.031 |
| X30 | 6.27 | 0.37 | 0.16 | -- | -- | -- |
| X31 | 6.63 | 0.28 | 0.13 | 0.25 | 0.06 | 0.005-0.06 |
| X32 | 7.04 | 0.90 | 0.36 | 0.38 | 0.092 | 0.005-0.092 |
| X33 | 7.60 | 0.55 | 0.53 | 0.32 | 0.077 | 0.077-0.17 |
| X34 | 8.26 | 1.44 | 0.47 | 0.49 | 0.118 | 0.118-0.17 |
| X35 | 8.77 | 0.90 | 0.47 | 0.46 | 0.111 | 0.111-0.17 |
| X36 | 9.19 | 0.72 | 0.55 | 0.27 | 0.065 | 0.065-0.17 |
| X37 | 9.83 | 1.00 | 0.54 | 0.31 | 0.075 | 0.075-0.17 |

# Supplementary Figures


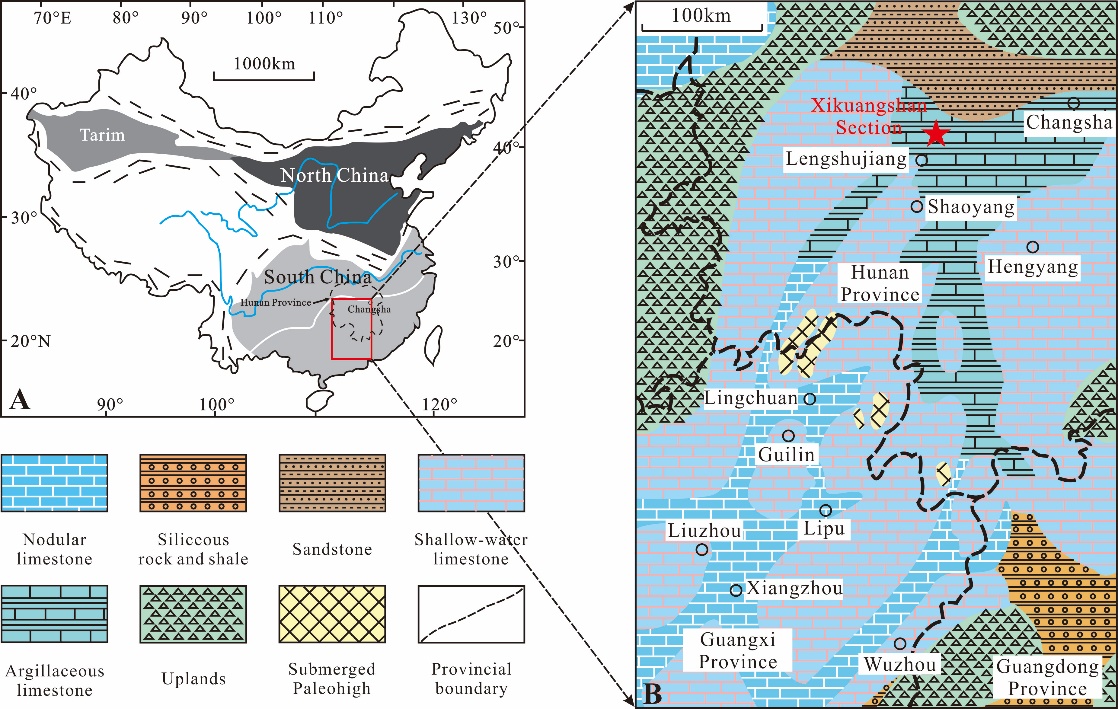


**Figure S1.** (A) Location map, and (B) paleogeographic map (modified from Chen et al. (15))


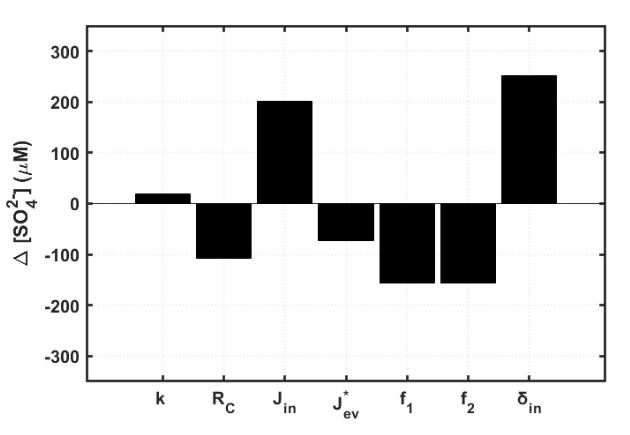


**Figure S2.** Change in seawater sulfate concentration in response to changes in isotope-driven model parameter values from lowest to highest values.


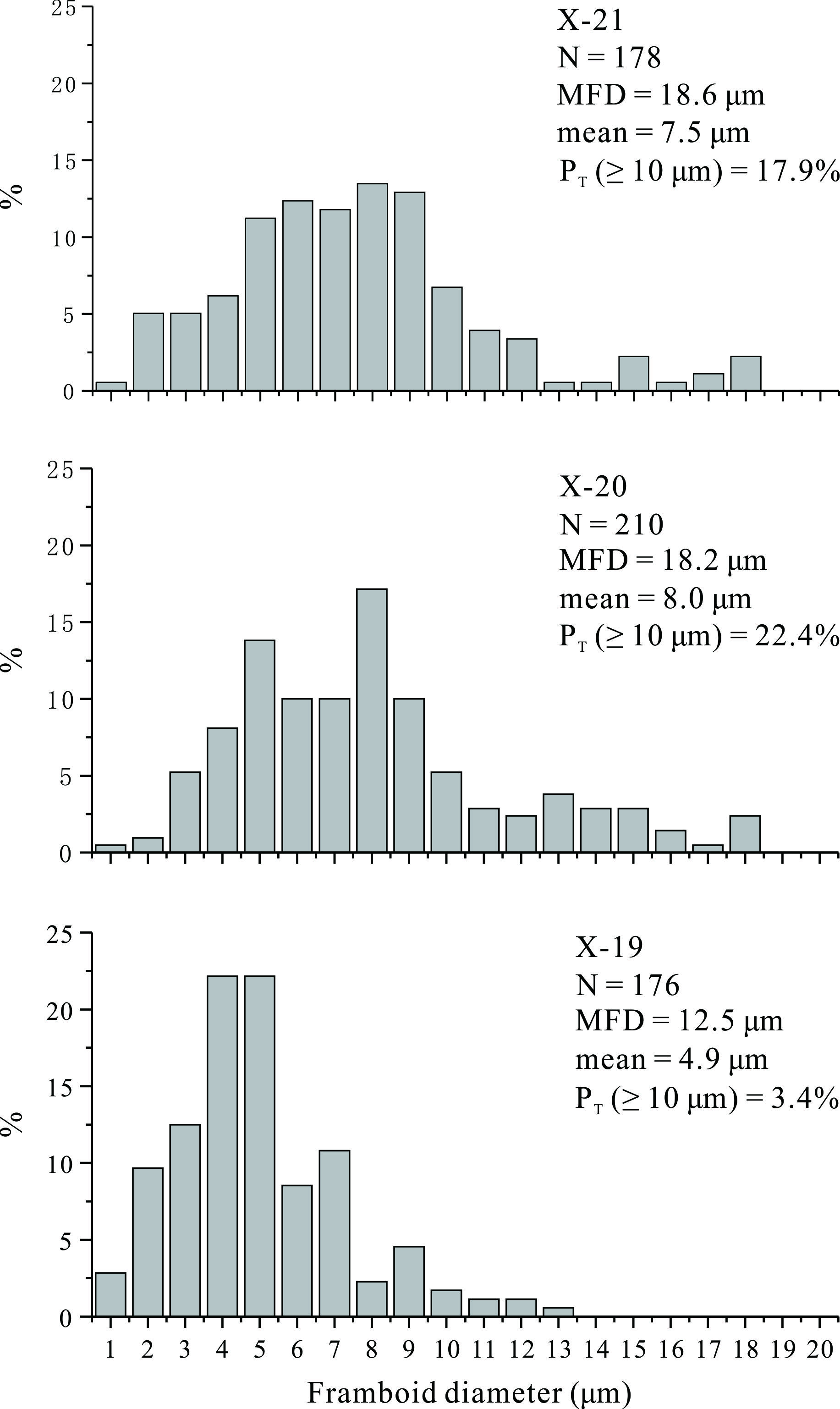


**Figure S3.** Histograms showing pyrite framboid size distributions from mudstone sample X-19 in the beginning of the UKH, indicating a euxinic environment, and samples X-20 and X-21 during the UKH.


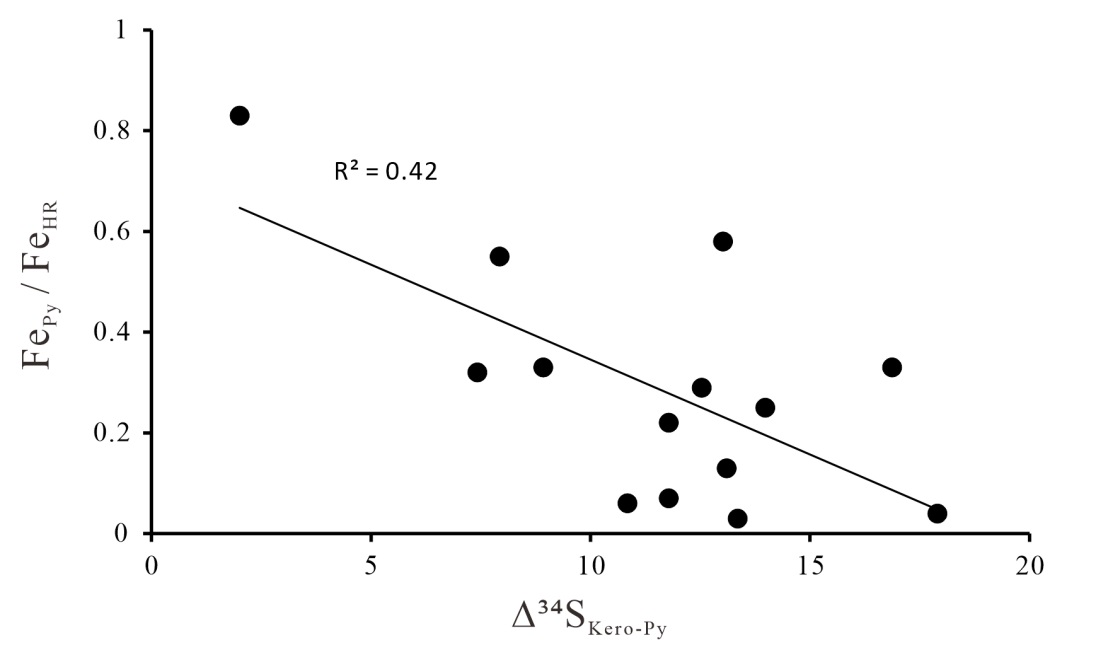


**Figure S4.** Cross plot of Fe_Py_/Fe_HR_ ratio with Δ^34^S_Kor-Py_ showing a negative relationship


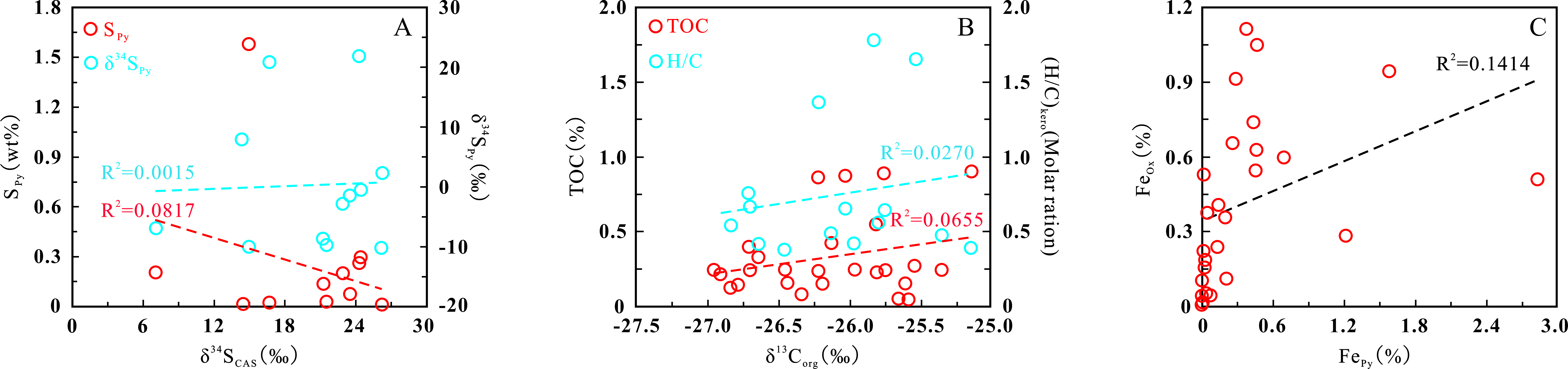


**Fig. S5** Poor relationships between S_Py_ content versus δ^34^S_CAS_ and δ^34^S_Py_ versus δ^34^S_CAS_ (A), between TOC versus δ^13^C_org_ and H/C molar ratios versus δ^13^C_org_ (B), and between Fe_Ox_ content versus Fe_Py_ content (C) at Xikuangshan section, suggesting that sulfur and organic carbon isotopic compositions and Fe speciation were not significantly altered by diagensis.


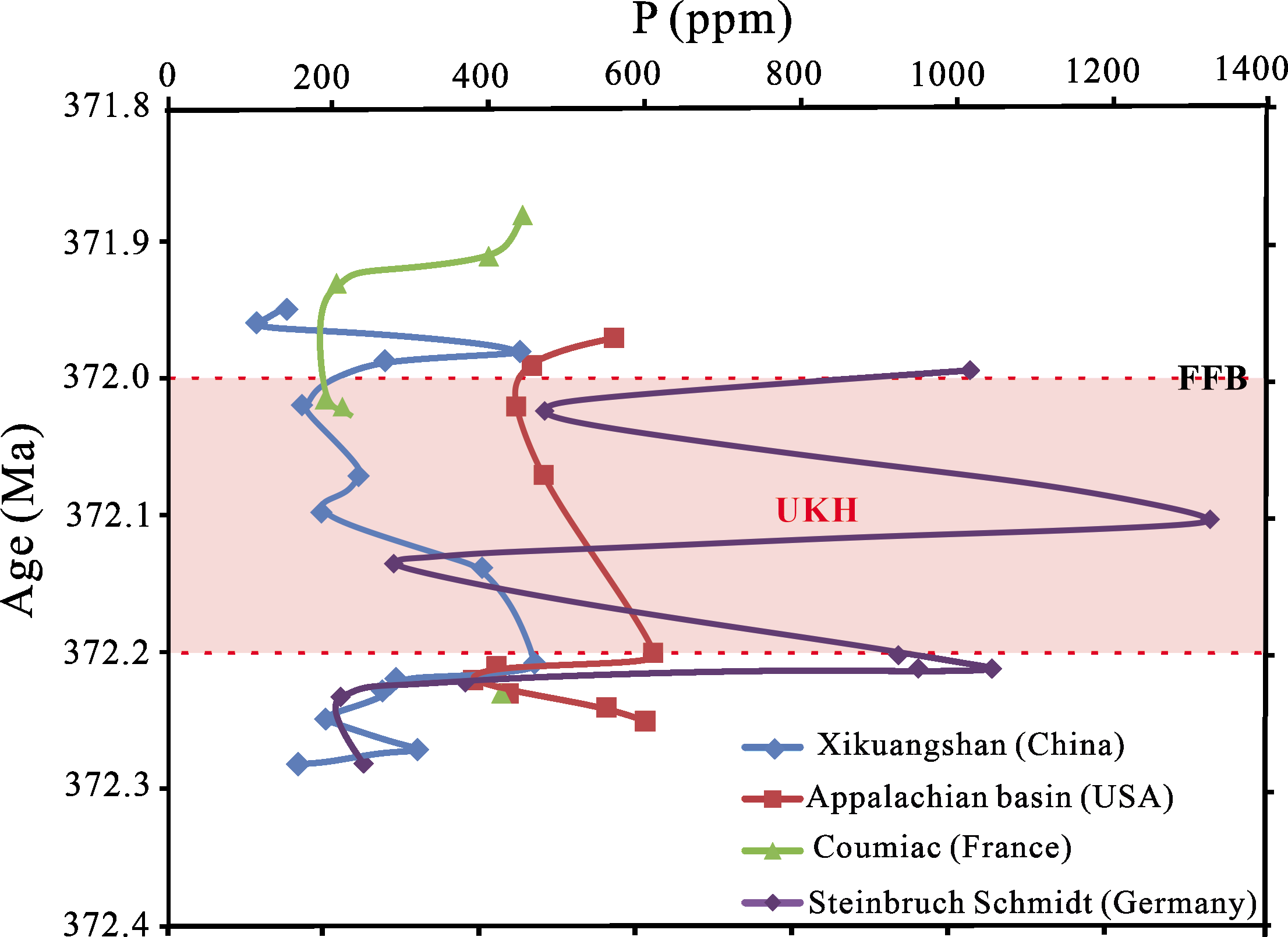


**Figure S6** Variation of P contents from worldwide sections with conodont zones and thickness corrected to Age (Ma). Data for Xikuangshan (China) are from this study, for Appalachian basin (USA) from Sageman et al. (54), and are enlarged for 5 times, and Coumiac (France) and Steinbruch Schmidt (Germany) from Percival et al. (55).
